# Mechanistic Characterization of a Small Molecule as a Direct NLRP3 Inhibitor via Binding to the NACHT Domain

**DOI:** 10.1021/acs.jmedchem.5c02163

**Published:** 2025-11-12

**Authors:** Yiming Xu, Hallie Blevins, Savannah Biby, Jannatun N. Namme, Kun Zhang, Renfeng Li, Shijun Zhang

**Affiliations:** † Department of Medicinal Chemistry, School of Pharmacy, 6889 Virginia Commonwealth University, Richmond, Virginia 23298, United States; ‡ Philips Institute for Oral Health Research, School of Dentistry, Virginia Commonwealth University, Richmond, Virginia 23298, United States; § Department of Microbiology and Molecular Genetics, 6614 University of Pittsburgh, Pittsburgh, Pennsylvania 15232, United States

## Abstract

The NLRP3 inflammasome has recently emerged as a viable drug target, and small-molecule inhibitors of this protein complex are being actively tested in preclinical disease models. However, the mechanisms of action for the majority of these compounds remain unclear. Our recent medicinal chemistry campaign led to the discovery of several potent lead NLRP3 inhibitors from a distinct chemical scaffold. Herein, further characterization using biophysical, biochemical, chemical biology, and molecular biology approaches of one of the lead inhibitors revealed direct binding to the NACHT domain of the NLRP3 protein. The studies also suggested potentially multiple binding sites within the NACHT domain that can be targeted by different small-molecule inhibitors. Furthermore, an activity-based probe with high labeling efficacy was developed and can serve as a valuable tool to contribute to ongoing efforts in understanding NLRP3 biology and in developing NLRP3-targeted therapies for various diseases.

## Graphical abstract

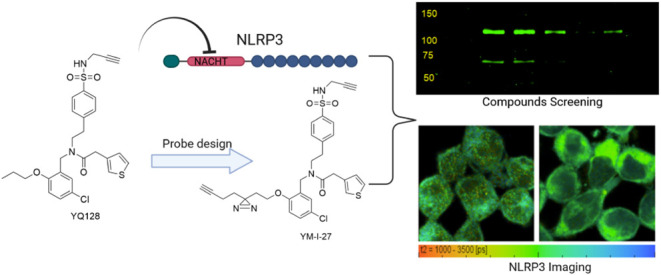

## Introduction

The NOD-like receptor (NLR) protein 3 (NLRP3) inflammasome is a cytosolic protein complex that plays an important and intricate role in regulating the innate immune responses by mediating the release of the pro-inflammatory cytokines interleukin (IL)-1β and IL-18.^1^ Architecturally, the inflammasome consists of the sensor protein NLRP3, the apoptosis-associated speck-like protein containing a caspase activation and recruitment domain (CARD) (ASC), and pro-caspase-1. The canonical activation of this protein complex involves a two-step process: priming and activation. The priming step upregulates the transcriptional expression of NLRP3, pro-caspase 1 and pro-IL-1β, whereas activation occurs following the recognition of related pathogen-associated molecular patterns (PAMPs) and/or damage-associated molecular patterns (DAMPs) through the sensor protein NLRP3 and induces inflammasome formation.^2,3^ Structural biology studies have revealed both the inactive and active forms of the NLRP3 protein, providing critical structural insights into the activation and assembly of the protein complex. The pathophysiological roles of aberrant NLRP3 inflammasome activation have been demonstrated in many human diseases, including atherosclerosis,^4,5^ diabetes,^6^ obesity,^7^ inflammatory bowel disease,^8^ gout,^9^ and neurodegenerative diseases.^10–13^ In the brain, dysregulation and overactivation of the NLRP3 inflammasome serve as an essential contributor to neuroinflammation, a common pathology shared by neurodegenerative disorders.^14^ For example, in Alzheimer’s disease, the pathological roles of the NLRP3 inflammasome have been demonstrated by genetic, pharmacological, and molecular studies in preclinical animal models or patient samples.^15,16^ Dysregulation of the NLRP3 inflammasome pathway is closely associated with other pathologies, e.g., Aβ and tau in AD, α-synuclein in Parkinson’s disease, ultimately, this leads to glia cell activation and neuronal damage.^15,17–20^ Collectively, this highlights the therapeutic potential of NLRP3-targeted compounds to achieve disease interventions for both peripheral and brain disorders.

Numerous small molecules from several chemotypes have been reported as direct or indirect NLRP3 inflammasome signaling inhibitors. Among them, MCC950,^21,22^ CY-09,^23^ tranilast,^24^ and oridonin^25^ target the NLRP3 protein by directly interacting with its NACHT domain of NLRP3. Recently, structural biology studies of NLRP3 in complex with MCC950 revealed that MCC950 forms interactions with multiple subdomains of the NACHT domain, thus keeping NLRP3 in an inactive state and preventing adenosine triphosphate (ATP) hydrolysis, ultimately inhibiting inflammasome assembly.^26^ This is instrumental in guiding the development of MCC950 analogs, addressing concerns such as off-target effects,^27^ toxicity,^28^ as well as limited brain penetration,^29^ which are the focus of the majority of the issued patents or primary literature publications.^30–34^ Some MCC950 chemotype-based NLRP3 inhibitors have entered the phase of clinical studies, including DFV-890, ZYIL-1, selnoflast, and emlenoflast, among others.^35–38^ NLRP3 inhibitors based on different chemical scaffolds have also been reported,^34,39–43^ and recently, a pyridazine-based inhibitor, AZD4144, developed by AstraZeneca, has entered Phase I clinical trials to assess its safety, tolerability, and pharmacodynamics (PD).^44^ Although significant advances have been made in the development of NLRP3 inhibitors, no drug has yet been approved by the FDA. Therefore, NLRP3 inhibitors featuring novel scaffolds and distinct mechanisms of action (MOAs) are still needed to fill the pipeline of NLRP3 inhibitor development. Recently, our group embarked on the development of a sulfonamide-containing scaffold as NLRP3 inhibitors, and some of the lead compounds exhibited promising *in vivo* efficacy in preclinical animal disease models.^45,46^ PET imaging studies employing radiotracers derived from the lead structures also demonstrated rapid brain uptake and specific binding of the NLRP3 protein in brain tissues.^47,48^ Herein, we report mechanistic studies using one of the lead compounds, YQ128 (Figure 1A), and probes derived from this compound to understand how our inhibitors interact with the NLRP3 protein. The results support a novel MOA for compounds derived from this sulfonamide-containing scaffold as direct NLRP3 inhibitors.

**Figure 1:**
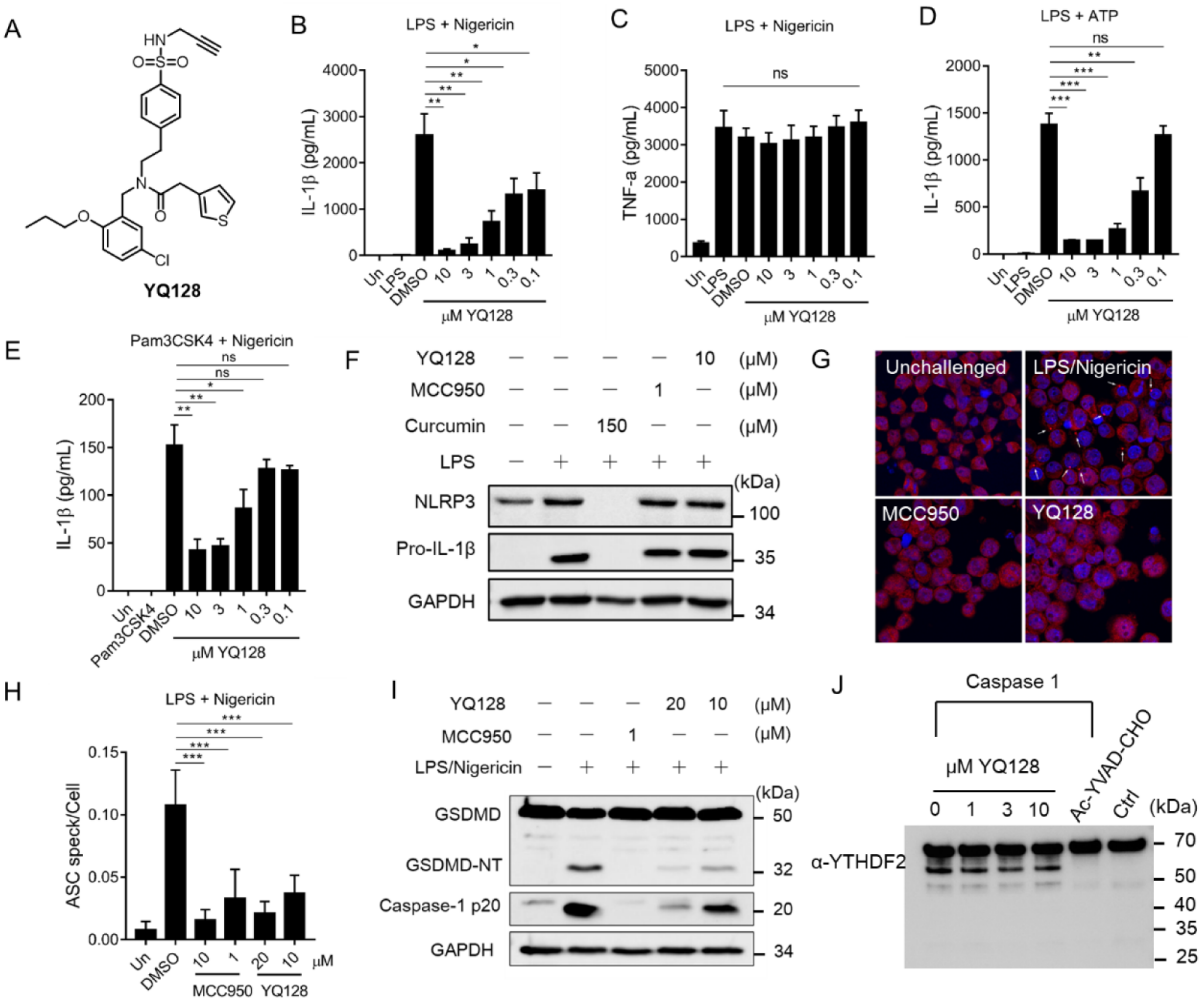
YQ128 inhibits the activation of the NLRP3 inflammasome. (A) Chemical structure of YQ128. (B–E) J774A.1 cells were primed with LPS (1 μg/mL) or Pam3CSK4 (400 ng/mL) and stimulated with nigericin (10 μM) or ATP (5 mM) with or without YQ128. The levels of IL-1β and TNF-α in the culture media were measured by enzyme-linked immunosorbent assay (ELISA). (F) Western blot analysis of NLRP3 and pro-IL-1β in cell lysates from J774A.1 cells treated with YQ128 (10 μM), curcumin (150 μM), or MCC950 (1 μM) and stimulated with LPS (1 μg/mL). (G) LPS-primed J774A.1 cells were treated with MCC950 (1 and 10 μM) or YQ128 (10 and 20 μM) before stimulation with nigericin and analyzed by Immunostaining (White arrows indicate ASC specks). (H) Quantitative analysis of the number of cells containing ASC specks. (I) Western blotting analysis of cleavage of caspase-1 and GSDMD in cell lysates from J774A.1 cells stimulated with LPS (10 μg/mL)/nigericin (10 μM). (J) Western blot analysis of cleavage of recombinant YTHDF2 by caspase-1 treated with YQ128 (1, 3, and 10 μM). Statistical analysis by unpaired Student’s *t*-test: *p < 0.05, **p < 0.01, ***p < 0.001, *ns* means not significant.

## Results and Discussion

### YQ128 Inhibits IL-1β Production through Interference with NLRP3 Inflammasome Activation

A plethora of stimuli can activate the NLRP3 inflammasome under both canonical and noncanonical conditions. As shown in Figure 1B–E, YQ128 dose-dependently suppressed the production of IL-1β upon stimulation with lipopolysaccharide (LPS) or Pam3CSK4 followed by nigericin or ATP in J774A.1 cells. IL-1β secretion in THP-1 cells and iBMDMs upon activation with LPS/nigericin can also be suppressed by YQ128 (Figure S1), supporting the notion that YQ128 functions as an inhibitor of the NLRP3 inflammasome under a broad spectrum of conditions. No inhibition was observed for the production of tumor necrosis factor (TNF)-α under LPS/nigericin conditions upon treatment with YQ128 (Figure 1C). Molecular biology studies by Western blotting in murine macrophage J774A.1 cells demonstrated that LPS priming significantly increased the expression of NLRP3 and pro-IL-1β, and curcumin, a known NF-κb pathway inhibitor,^49^ abolished the expression of both proteins as expected. Notably, treatment with neither YQ128 nor MCC950 changed the levels of LPS-induced pro-IL-1β and NLRP3 (Figure 1F), suggesting no effect of YQ128 on the LPS-induced priming stage. Similar effects were observed in THP-1 cells under the same conditions (Figure S1). Immunofluorescence studies demonstrated that treatment of murine macrophage J774A.1 cells with YQ128 significantly suppressed the formation of ASC specks, a hallmark of NLRP3 inflammasome activation, upon LPS/nigericin activation (Figure 1G,H). The unchallenged group here served as a negative control, in which no ASC specks are expected. Studies have demonstrated that potassium channel blockade led to the formation of ASC specks and inflammasome activation.^50,51^ The observed inhibitory effect of YQ128 on ASC speck formation thus hints that upstream interference on the potassium channels by YQ128 might not be a contributing factor for the observed inhibitory effects of YQ128 under the tested conditions. Furthermore, treatment with YQ128 in J774A.1 cells under LPS/nigericin conditions showed no significant effect on the production of mitochondrial ROS and integrity (Figure S2). Further analysis of the cleavage of caspase-1 and the immediate downstream substrate of the NLRP3 inflammasome, gasdermin D (GSDMD), revealed an inhibitory effect by YQ128 treatment for both events (Figure 1I). Biochemical studies using recombinant caspase-1 and a peptide substrate demonstrated that YQ128 does not interfere with the catalytic activity of caspase-1 (Figure 1J). Furthermore, the selective inhibition of NLRP3 inflammasome activation by YQ128, but not NLRC4 and AIM2 inflammasomes, indicates no involvement of downstream GSDMD-mediated pathways.^52^ Collectively, the results shown here strongly suggest that the observed inhibition of IL-1β production by YQ128 occurs via direct inhibition of the NLRP3 inflammasome complex.

### YQ128 Binds to the NLRP3 Protein

During the assembly of the NLRP3 inflammasome complex, multiple components, including NLRP3, NIMA-related kinase 7 (NEK7), and ASC are involved through oligomerization and recruitment. We hypothesize that YQ128 interferes with inflammasome activation by interacting with at least one of these proteins. To identify the potential binding partner(s), a photoaffinity labeling (PAL) approach was attempted. Our previous structure–activity relationship (SAR) studies suggested that chemical modifications on the propoxy moiety of the YQ128 scaffold can be tolerated and have minimal impact on the biological activities.^52^ Therefore, as shown in Figure 2A, the PAL probe, YM-I-85, was designed to incorporate a diazirine moiety as the photoreactive group and a biotin moiety as an enrichment handle using streptavidin resin into the structure at the propoxy position. After chemical synthesis (Supplementary schemes 1–5), biological characterization in J774A.1 cells confirmed its inhibitory potency on the production of IL-1β upon LPS/ATP activation with an IC_50_ of 2.4 μM. Although its potency is ∼8-fold less than that of YQ128, given that this probe will be used with cell lysates, the potency and membrane penetration should not be limiting factors here. As shown in Figure 2B, PAL and pull-down studies using cell lysates of LPS-primed J774A.1 cells upon incubation with YM-I-85 followed by UV irradiation at 360 nm wavelength revealed that YM-I-85 efficiently labeled NLRP3 and NEK7 in a dose-dependent manner but not ASC. We then rationalized that the specific binding site of this probe within the NLRP3 protein will be mostly occupied upon pretreatment of the LPS-primed J774A.1 cell lysates with the parent compound YQ128; therefore, the interaction and labeling efficiency of NLRP3 with YM-I-85 will be reduced. Notably, YQ128 blocked the interaction of YM-I-85 with NLRP3, as expected, but not with NEK7, supporting the specific interaction of YM-I-85 with NLRP3. To confirm the interaction of YQ128 with NLRP3, a cellular thermal shift assay (CETSA)^53^ using Expi293F cells expressing Flag-NLRP3 in the presence and absence of YQ128 was performed. The results in Figure 2C revealed that the presence of YQ128 increased the stability of NLRP3 during thermal denaturation from 45 to 67 °C, hinting at the direct interaction of YQ128 with NLRP3. Microscale thermophoresis (MST) studies using recombinant human NLRP3 protein also supported the binding of YQ128 to NLRP3 with a *K*_d_ of 117 nM (Figure 2D).

**Figure 2:**
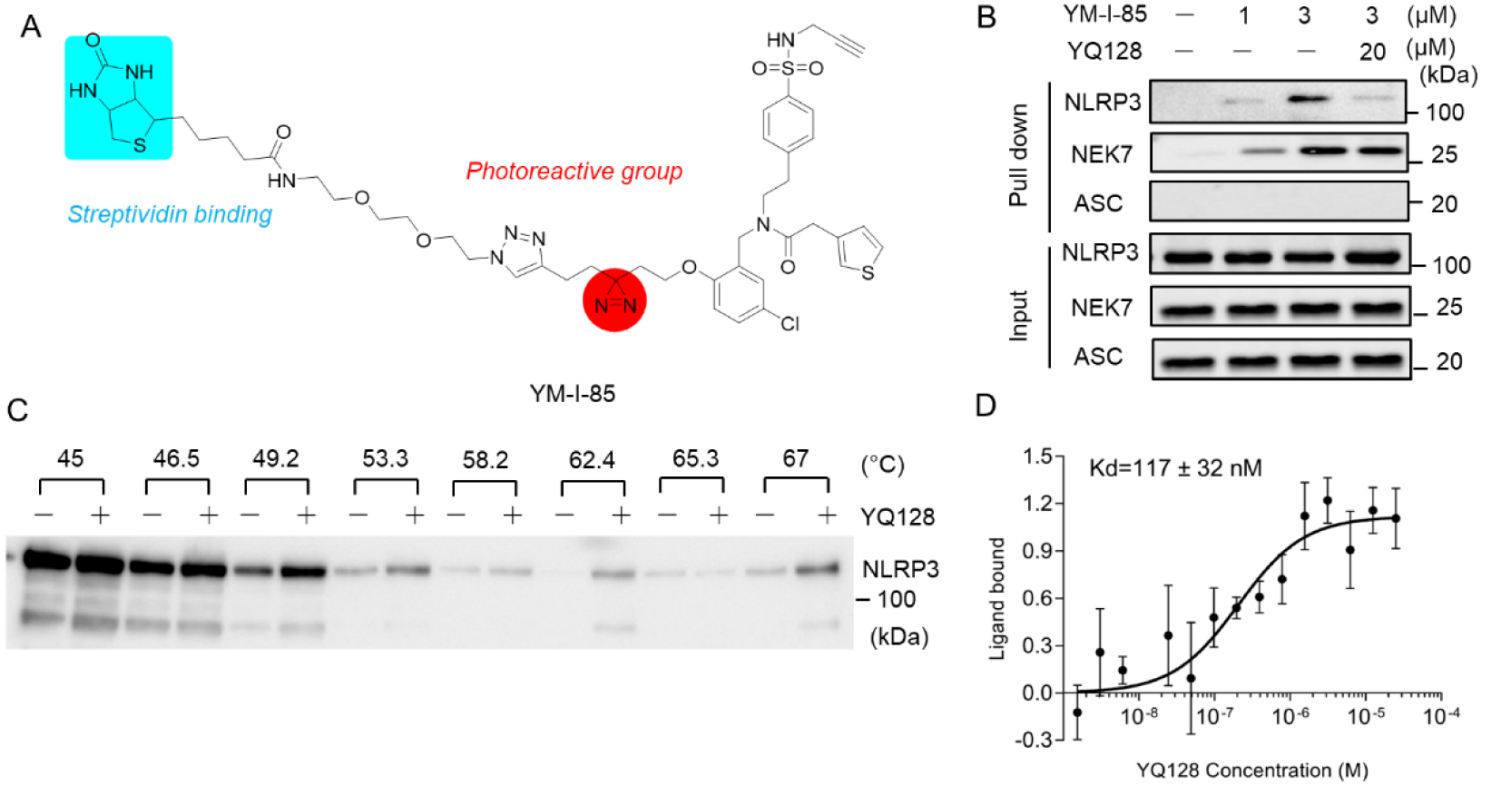
YQ128 binds to NLRP3. (A) Chemical structure of YM-I-85. (B) Western blot results of the pull-down study of YM-I-85 from J774A.1 cell lysate primed with LPS (1 μg/mL) under the indicated conditions. (C) CETSA analysis in Expi293F cells expressing Flag-NLRP3 in the presence/absence of YQ128 (50 μM). (D) Binding affinity of YQ128 to the recombinant human NLRP3 protein was measured by an MST assay.

### YQ128 Binds to the NLRP3 Protein via the NACHT Domain

To better understand which domain of NLRP3 YQ128 binds, drug affinity responsive target stability (DARTS) experiments^54^ were conducted using full-length NLRP3, the pyrin domain (PYD)-NACHT fragment, and the PYD fragment (Figure 3A). As shown in Figure 3B–D, the digestion pattern of the full-length NLRP3 and the PYD-NACHT fragment, but not the PYD fragment, by Pronase was altered in the presence of YQ128. Further analysis of the fragmentation of the full-length NLRP3 and PYD-NACHT also showed increased stability of fragments between 25 and 50 kDa in the presence of YQ128. Again, no significant difference was observed for the PYD fragment in this region, regardless of the presence or absence of YQ128. The results suggest that YQ128 might bind to the NLRP3 protein via its interactions with the NACHT domain. With MCC950 as a positive control, a binding *K*_d_ of 165 nM between YQ128 and the NACHT protein, determined by surface plasmon resonance (SPR), provided further evidence for the binding interactions of YQ128 with the NACHT domain (Figure 3E,F), thus supporting and validating the results from the DARTS experiments. This notion is further supported by the results from dot blot analysis. Both MCC950 and YQ128 dose-dependently decreased the recognition of NLRP3 by the D4D8T antibody, which has an epitope at Ala306 within the NLRP3 NACHT domain (Figure S3).

**Figure 3:**
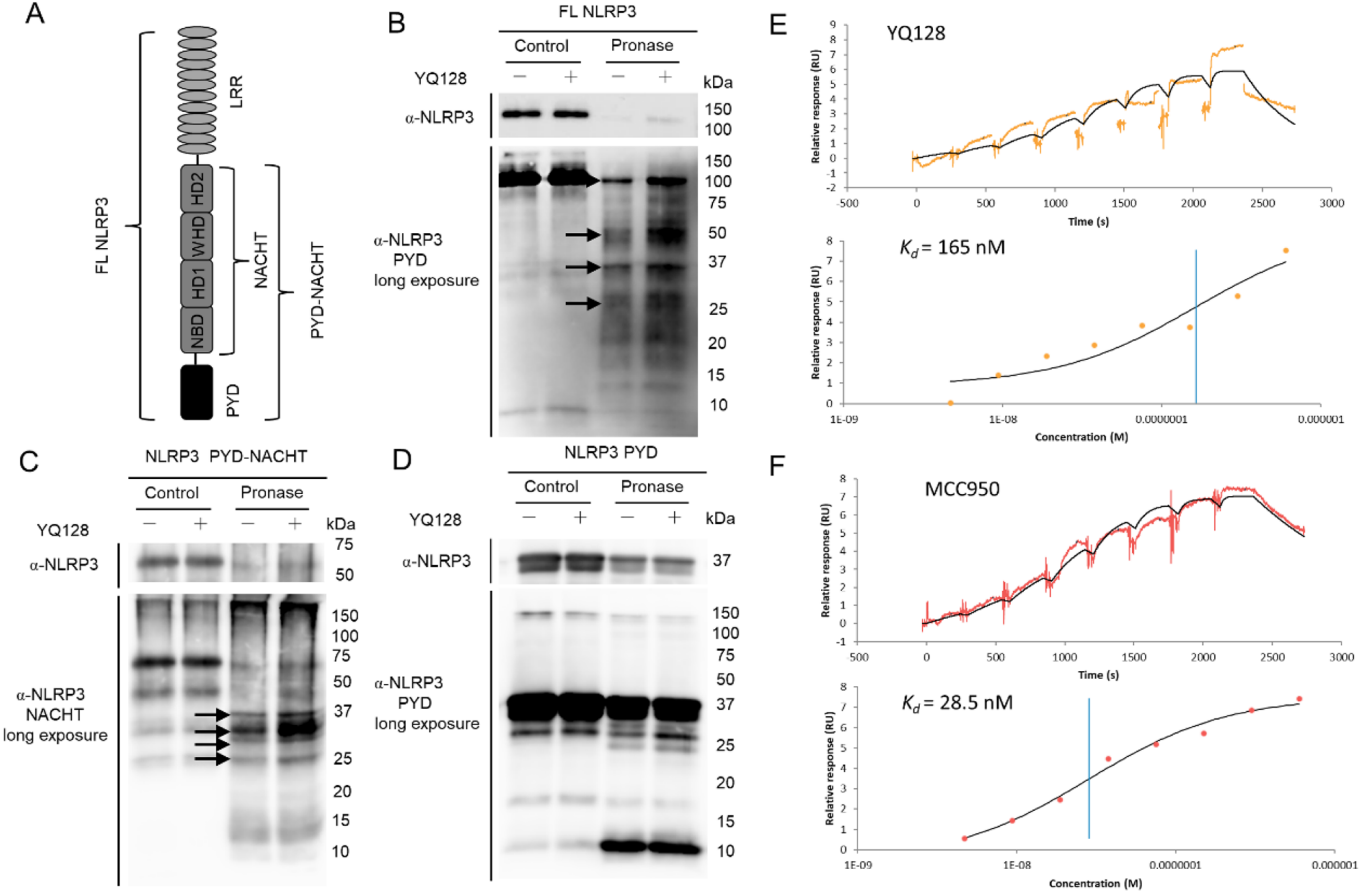
YQ128 interacts with the NACHT domain of NLRP3. (A) Schematic diagram of the domains of the NLRP3 protein. Recombinant full-length (FL) human NLRP3 (B), PYD-NACHT domain (C), and PYD domain (D) were digested with Pronase (stock: 0.1 mg/mL) at 50–200 ng of Pronase per μg of protein ratio, with or without YQ128 (50 μM), and analyzed by Western blotting using corresponding antibodies (Cryo-2 antibody for FL NLRP3 and PYD domain; D4D8T antibody for PYD-NACHT domain). Arrows indicate changes observed with YQ128 treatment. YQ128 (E) and MCC950 (F) bind to the NLRP3 NACHT domain, as measured by SPR.

### A YQ128-Based, Cell-Permeable Probe Binds to NLRP3 In Vitro

Recently, fluorescent dye- or biotin-conjugated MCC950 and CY-09 probes (Figure 4) have been developed and reported as tools to facilitate drug discovery efforts. For example, probes 1 and 2 were developed for imaging purposes; probe 3 was used for NanoBRET experiment; and probe 4 was developed for a fluorescence polarization (FP) assay.^55–58^ Given that our results strongly suggest YQ128 as a selective and direct NLRP3 inhibitor, we sought to modify it to create a probe with flexibility for different uses. Therefore, YM-I-27 (Figure 5A) was designed with the following reasons: (1) two alkyne moieties are incorporated and this will allow for efficient click-chemistry to add fluorescent tags or biotin handles. But we do not expect any reaction specificity of the alkyne handles to either fluorescent or pull-down tags; (2) a diazirine moiety is introduced to allow formation of stable and covalent adducts with interacting proteins upon UV activation, and this will provide tolerability in the following experimental handling; and (3) compared to the probes shown in Figure 4, we envision that YM-I-27, with a much shorter side chain and smaller size, would have better membrane permeability that allows for its use as a tool for studies *in situ* and in live cells.

**Figure 4:**
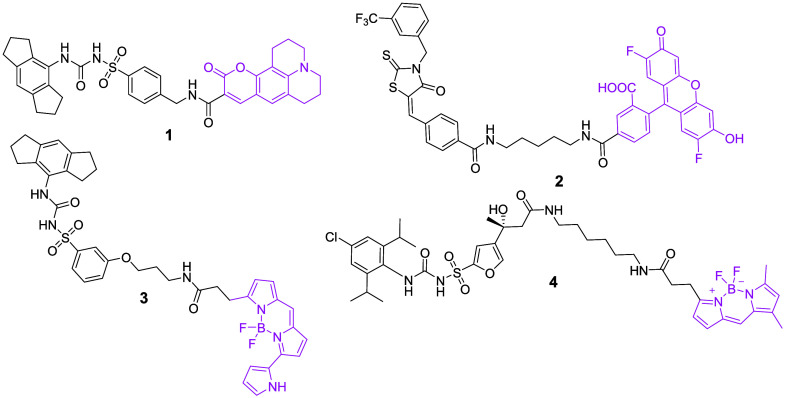
Chemical structures of the recently reported fluorescent and biotin-conjugated probes derived from MCC950 and CY-09.

**Figure 5:**
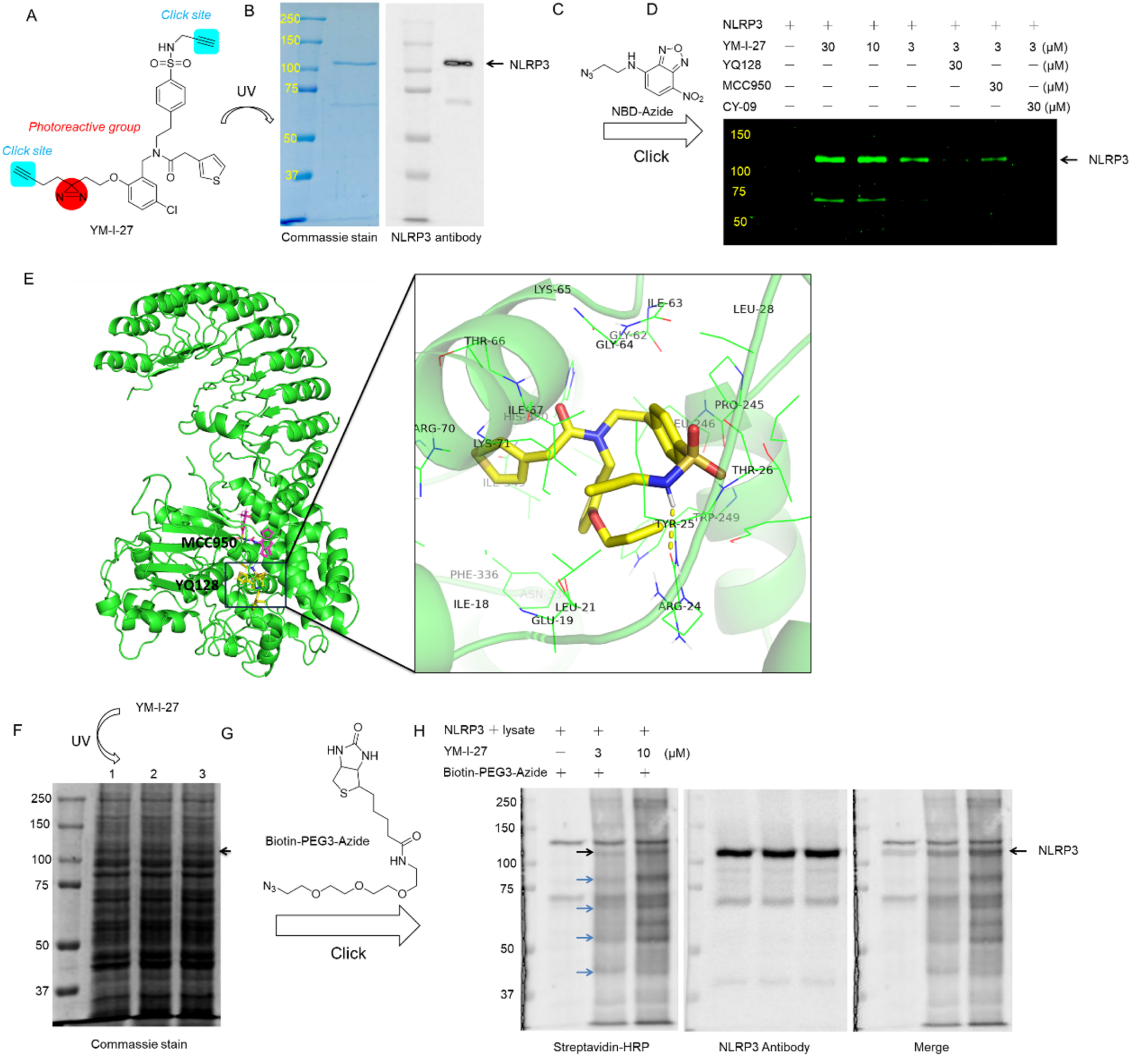
YM-I-27 efficiently labels NLRP3. (A) Chemical structure of YM-I-27. (B) Confirmation of purified mouse Flag-NLRP3 protein. (C) Chemical structure of NBD-Azide. (D) Cross-linking of YM-I-27 with recombinant NLRP3 using click reaction agents (stock solution: 5 mM NBD-Azide, 50 mM CuSO_4_, 30 mM TBTA, and 100 mM TCEP) with or without YQ128 under the indicated conditions. (E) Superimposed images of YQ128 and MCC950 binding to their respective binding sites and predicted binding model of YQ128 with NLRP3 (PDB: 7PZC). (F) Three aliquots (lanes 1–3) of J774A.1 cell lysate spiked with NLRP3 at quality ratio of 20:1 and Coomassie staining results. The samples were then used for proteome click labeling. (G) Chemical structure of Biotin-PEG3-Azide. (H) *In situ* proteome click labeling using YM-I-27 (3 and 10 μM) and click reaction agents (stock solution: 5 mM Biotin-PEG3-Azide, 50 mM CuSO_4_, 30 mM TBTA, and 100 mM TCEP). The results were analyzed by immunoblotting using Streptavidin-HRP (1:1,000) and NLRP3 antibodies (D4D8T (1:1,000)). Black arrows indicate NLRP3 protein; blue arrows indicate other potential cellular targets of YM-I-27.

Notably, YM-I-27 showed potent inhibition of IL-1β production following LPS/ATP activation in J774A.1 cells, with an IC_50_ of 0.5 μM, comparable to that of YQ128. This may further ensure its potential as a chemical probe for *in vitro*, cell-based studies. Recombinant NLRP3 was first preincubated with a range of compounds, including YQ128, MCC950, and CY-09, to evaluate competitive binding, and was subsequently treated with YM-I-27. Following UV-activated cross-linking, a fluorescent nitrobenzoxadiazole (NBD) moiety (Figure 5C) was introduced to the probe for fluorescent visualization by reacting NBD-N_3_ with the alkyne moiety within the probe via Cu (I)-catalyzed click chemistry. As shown in Figure 5D, YM-I-27 dose-dependently labeled the NLRP3 protein, and YQ128 outcompeted the labeling, suggesting its specific interactions with NLRP3. MCC950, a tight binder of NLRP3, only partially blocked the labeling of YM-I-27 under the current experimental condition. This is consistent with the results from the Promega studies in their NanoBRET target engagement assay study using a MCC950-based probe, which showed weak competition by YQ128.^57^ This was further confirmed by the results from an FP assay (Figure S4). The results may suggest that, even though both compounds bind to the NACHT domain of NLRP3, they have different binding sites within this domain. Further studies, such as structural biology investigations, are warranted to provide definitive evidence for the binding site of the compound. However, the current preliminary results from well-established experimental methods and protocols strongly suggest the existence of multiple binding sites within the NACHT domain that can be targeted by small-molecule inhibitors. The results also highlight that YM-I-27 could serve as a new NLRP3-targeting probe to facilitate future mechanistic studies and drug discovery efforts. CY-09 completely blocked the interaction of YM-I-27 with the protein under the current experimental conditions. Studies have shown that MCC950, as an allosteric inhibitor, binds to the bottom of the central cavity formed by the subdomains of WHD, HD1, NBD, HD2, and LRR within the NACHT domain of NLRP3, while CY-09 interacts with the ATP-binding site.^23,26^ The results from the PAL studies may suggest that YM-I-27 also interacts with the ATP-binding site. To better understand how YQ128 interacts with the NLRP3 protein, molecular docking studies using the reported NLRP3 structure (PDB: 7PZC) were performed, and the results revealed that YQ128 scored better (−8.26) with a glide energy of −58.054 within the ATP binding site compared to those of −6.505 (docking score) and −44.078 (glide energy) within the MCC950 binding site. As shown in Figure 5E, the 5-chloro-2-propoxybenzyl moiety and the thiophen moiety of YQ128 form hydrophobic interactions within the ATP binding pocket. In addition, a H-bond interaction was observed between the sulfonyl amide moiety and ARG24 (2.2 Å). Overall, YQ128 adopted a T-shaped structure under this binding mode and blocks access of ATP to the binding cavity. Overall, the docking results suggest that YQ128 fits favorably to the ATP-binding site, which is consistent with our experimental data.

We next performed *in situ* proteome labeling using J774A.1 cell lysates (Figure 5F) to shed light on the potential cellular targets of this probe and its labeling efficiency. To this end, we intended to conjugate a biotin tag to the probe to allow cross-validation of protein labeling by Western blotting analysis. The initial screening results revealed that Biotin-PEG3-N_3_ (Figure 5G) is able to efficiently form conjugates with the probe after cross-linking. As shown in Figure 5H, the results suggested that YM-I-27 successfully and efficiently recognized NLRP3 along with four other proteins.

### YM-I-27 Colocalizes with NLRP3 in Live Cells

To further evaluate the mode of localization of this probe and whether it interacts with endogenous NLRP3 in a cellular environment, we designed an immunocytochemistry fluorescence resonance energy transfer (FRET) assay, as illustrated in Figure 6A, by taking advantage of the probe’s permeability properties, its covalent cross-linking with interacting partners, and its ability to conjugate with a fluorescent tag. Alexa Fluor 488 (499/520 nm ex/em) and TAMRA (564/579 nm ex/em) were selected as the donor/acceptor pairs in this experiment. The analysis of the FRET donor’s fluorescence lifetime distribution (Figure 6B,C) showed that the emission lifetime of Alexa Fluor 488 was significantly decreased by ∼ 44 ps in the presence of YM-I-27, suggesting binding interactions of YM-I-27 with endogenous NLRP3, consistent with the results from the PAL studies.

**Figure 6:**
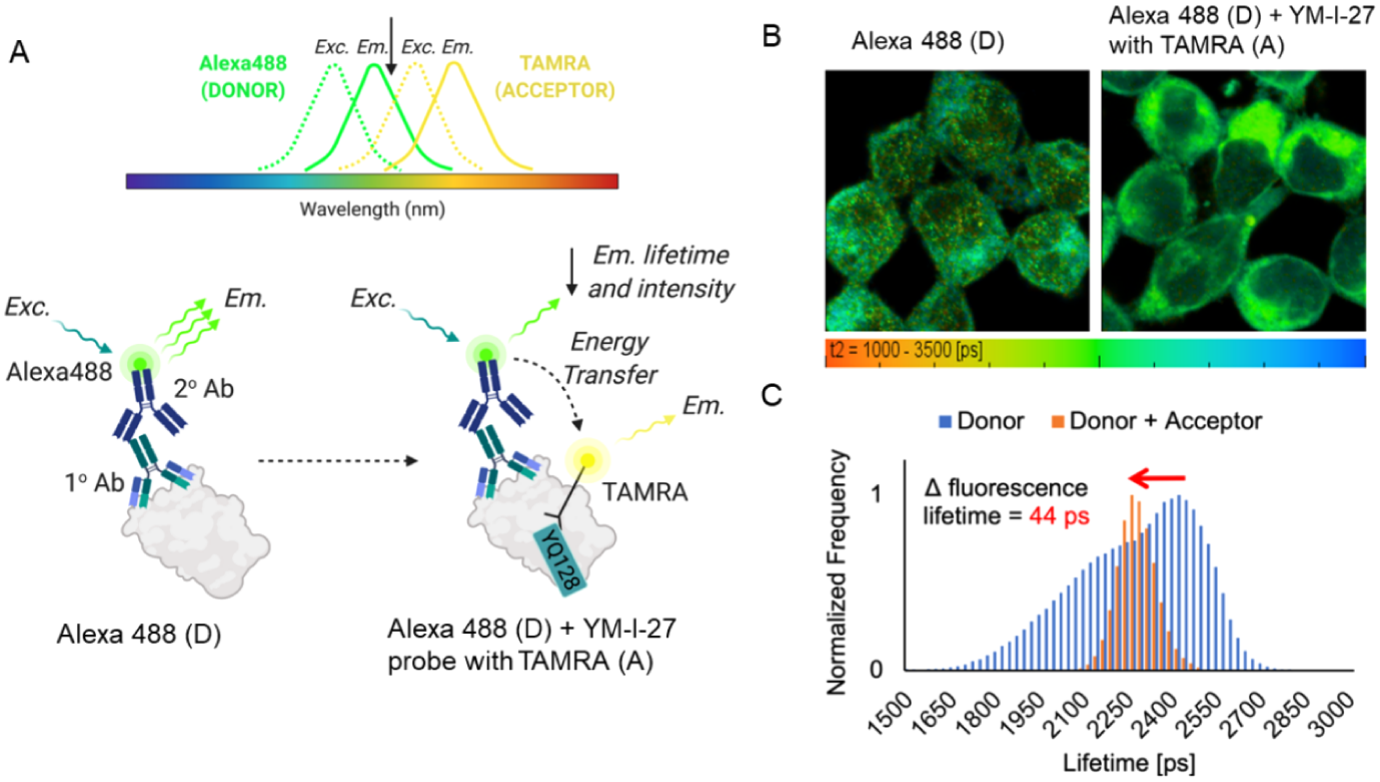
Co-localization of YM-I-27 with endogenous NLRP3 in live cells. (A) Schematic illustration of the designed FRET experiment. (B) Representative confocal microscopic images after acquisition by the acceptor (TAMRA labeled YM-I-27). (C) Analysis of the FRET donor’s fluorescence lifetime distribution.

## Conclusion

The NLRP3 inflammasome has emerged as an attractive drug target due to its involvement in the pathogenesis of immune and inflammatory disorders. Consequently, small molecule inhibitors of this protein complex have been actively pursued to achieve effective disease interventions. Although numerous inhibitors have been reported by targeting this protein complex and the signaling pathway associated with it, only a few of them have defined MOAs and are mainly associated with the MCC950 scaffold. Therefore, the development of NLRP3 inhibitors with distinct MOAs would be of significance to expand the pipeline and add chemical tools to better understand the biology of the NLRP3 inflammasome. Our recent medicinal chemistry campaign led to the discovery of a sulfonamide-containing scaffold as potent and selective NLRP3 inhibitors. Further *in vivo* studies in preclinical disease models and PET/CT imaging studies using radiotracers derived from the lead compounds demonstrated rapid brain penetration and *in vivo* efficacy.^48,52^ To better understand the MOA of this scaffold and guide further development of new analogs as NLRP3 inhibitors, one of the lead compounds, YQ128, was selected for detailed mechanistic studies in this report. The results from cellular assays under different activation conditions suggested that YQ128 suppresses the production of IL-1β by directly interfering with the protein complex. Further characterization using biochemical, biophysical, and PAL approaches suggested that YQ128 interacts with the NACHT domain of the NLRP3 protein, which is a common domain targeted by other reported NLRP3 inhibitors as well. The unique structure of YQ128 led us to design and develop an activity-based probe with the flexibility and versatility for *in situ* and *in vitro* studies, tailored through click chemistry. Notably, results from the PAL and *in situ* proteome labeling studies indicated that NLRP3 inhibitors based on this sulfonamide-containing scaffold might have a distinct binding site within the NACHT domain from those engaged by MCC950 or CY-09. More importantly, the results strongly suggest the existence of multiple binding sites within the NACH domain that may be exploited therapeutically. Therefore, our studies herein highlight a new class of NLRP3 inhibitors with a potential novel MOA, and the chemical probes derived from the lead compound YQ128 could serve as valuable pharmacological tools to expand our understanding in the pathophysiological roles of the NLRP3 inflammasome and facilitate drug discovery endeavors.

## Experimental Section

### Chemistry

All chemical reagents and solvents were obtained from commercial suppliers and used without further purification. Silica gel flash chromatography (200–300 mesh) was purchased by Fisher Scientific. The ^1^H and ^13^C nuclear magnetic resonance (NMR) spectra of compounds were collected using a Bruker ARX 400 spectrometer with CDCl_3_ or DMSO-*d*_6_ as the solvent. All chemical shifts (δ) are assigned as parts per million (ppm) using tetramethylsilane (TMS) as the internal standard. Signals are described as s (singlet), d (doublet), t (triplet), q (quartet), or m (multiplet). The purity of target compounds was determined to be ≥95% by HPLC using a Varian 100–5 C18 250 × 4.6 mm column with UV detection at 288 nm (70% acetonitrile/H_2_O with 0.1% TFA or 50% MeOH/H_2_O with 0.1% TFA). High-resolution mass spectrometry (HRMS) was performed on an Applied BioSystems 3200 Q trap with a turbo V source for Turbolon Spray. The detailed synthetic procedures are accessible in the Supporting Information.

### Cells

J774A.1 murine macrophage cells and THP-1 human monocytic cells were purchased from the American Type Cell Culture (ATCC, Manassas, VA). iBMDMs were obtained from BEI Resources. J774A.1 cells and iBMDMs were cultured in Dulbecco’s modified Eagle medium (DMEM) supplemented with 10% fetal bovine serum (FBS) and 1% penicillin/streptomycin at 37 °C and 5% CO_2_. THP-1 cells were cultured in RPMI 1640 supplemented with 10% FBS, 1% P/S, and 50 μM 2-mercaptoethanol at 37 °C and 5% CO_2_. Expi293F cells were purchased from Thermo Fisher Scientific (Waltham, Massachusetts) and were cultured in Expi293 Expression Medium and grown at 37 °C with 8% CO_2_ while being shaken at 125 rpm.

### Reagents and Antibodies

Adenosine triphosphate (ATP, A2383), Phorbol 12-myristate 13-acetate (PMA), paraformaldehyde (PFA), and 4’,6-diamidino-2-phenylindole (DAPI) were purchased from Millipore Sigma. Pam3CSK4 (tlrl-pms) and ultrapure LPS were purchased from InvivoGen. Nigericin (ab120494) was purchased from Abcam. Mouse IL-1β and TNF-α ELISA kits were obtained from R&D Systems. Ac-YVAD-CHO was obtained from VWR. NLRP3 antibody (Cryo-2) (1:1,000) and ASC antibody (AL177) (1:1,000) were purchased from AdipoGen. NLRP3 antibody (D4D8T) (1:1,000), gasdermin D antibody (E4M2W) (1:1,000), caspase-1 antibody (E2G2I) (1:1,000), GAPDH antibody (14C10) (1:1,000), and streptavidin-HRP antibody (3999) (1:2,000) were obtained from Cell Signaling. Secondary HRP-conjugated antibodies used were antirabbit IgG (7074) (1:1,000) from Cell Signaling.

### Activation of the NLRP3 Inflammasome

To induce inflammasome activation, J774A.1 cells (1 × 10^6^ cells/mL) were seeded in 96-well plates overnight. The medium was replaced the following day, and the cells were stimulated with LPS (1 μg/mL) or Pam3CSK4 (400 ng/mL) in DMEM for 4.5 h. The cells were treated with varying concentrations of YQ128 for 30 min and then stimulated with nigericin (10 μM) for 1 h or with ATP (5 mM) for 30 min. For iBMDMs, cells (1 × 10^6^ cells/mL) were seeded in 96-well plates overnight, then primed with LPS (1 μg/mL) for 3 h and stimulated with nigericin (10 μM) for 2 h. THP-1 cells were first differentiated into adherent macrophages by culturing for 24 h in complete RPMI 1640 medium containing 50 nM PMA, followed by a 24-h resting period in growth medium without PMA. The differentiated THP-1 cells were cocultured with LPS (1 μg/mL) for 2.5 h and stimulated with nigericin (10 μM) for 1 h. The levels of IL-1β and TNF-α in the supernatant were measured by ELISA.

### Fluorescence Microscopy

J774A.1 cells (5 × 10^5^ cells/mL) were plated in 12-well plates precoated with poly-l-lysine overnight. Then, J774A.1 cells were primed with LPS (1 μg/mL) for 4.5 h, stimulated with nigericin (3 μM), and stained with MitoSOX (5 μM) and MitoTracker Red (50 nM). The supernatants were discarded, and the cells were washed with ice-cold PBS three times and fixed with 4% PFA in PBS for 15 min. DAPI (5 μg/mL) was used for nuclear staining. After that, the cells were washed with PBS three times, and fluorescence microscopy analyses were carried out using an Olympus IX73.

### Immunoblotting Assay of the Activation of the NLRP3 Inflammasome

J774A.1 cells were seeded into a 24-well plate at 0.6 × 10^6^ cells/well and allowed to attach overnight at 37 °C. For the priming, cells were treated with compounds for 1 h, followed by 1 μg/mL LPS treatment for 4.5 h. Cells were lysed in RIPA buffer, and cell lysates were analyzed by immunoblotting. For the activation, cells were treated with 10 μg/mL LPS for 4.5 h. Compounds were added during the last hour of LPS treatment. Then, cells were treated with 10 μM nigericin for 1 h. Cells were lysed in RIPA buffer, and cell lysates were analyzed by immunoblotting.

### *In Vitro* Caspase-1 Cleavage Assay

The expression and purification of Halo-V5-YTHDF2 protein were performed as previously described.^59,60^ In brief, the Halo-tagged YTHDF2 plasmid was transfected into 293T cells at 50–60% confluence. After 48 h, two T175 flasks of transfected cells were harvested and lysed in 25 mL of HaloTag Protein Purification Buffer (50 mM HEPES, 150 mM NaCl, 1 mM dithiothreitol (DTT), 1 mM EDTA, and 0.005% NP40/IGEPAL CA-630, pH 7.5) supplemented with a Protease Inhibitor Cocktail. The Halo-tagged protein was then purified using Halo-tag resin, followed by elution with 3 × 0.5 mL of HaloTag Protein Purification Buffer containing 20 μL of Halo-TEV protease. The purified YTHDF2 was stored at −80 °C for further use.

Purified V5-YTHDF2, caspase-1, compound YQ128, and caspase-1 specific inhibitor Ac-YVAD-CHO (50 μM) were incubated in a caspase assay buffer (50 mM HEPES, 50 mM NaCl, 0.1% CHAPS, 10 mM EDTA, 5% glycerol, and 10 mM DTT, pH 7.2) at 37 °C for 2 h with gentle agitation. The reactions were then terminated by boiling in 2× SDS sample buffer, and the samples were analyzed by Western blotting.

### Recombinant NLRP3 Protein Expression and Purification

The recombinant full-length NLRP3 protein was expressed and purified as previously described using Expi293F cells.^47,61^ Briefly, the cells were transfected with 10His-hNLRP3–6His_pcDNA3.1 or pcDNA3-FLAG-mNLRP3 (Addgene no. 75127) using the ExpiFectamine 293 Transfection Kit (Thermo Fisher Scientific) following manufacturer’s instructions. Cells were harvested after 48 h, lysed, and centrifuged. Recombinant NLRP3 proteins were purified from supernatants with Nickel resin or M2 Anti-FLAG beads (Sigma-Aldrich), respectively, and then stored in 10% glycerol at −80 °C. Human biotinylated-NACHT domain of NLRP3 was expressed in Sf21 insect cells using the pFastBac1–8His-Strep II-ZZ-TEV-NLRP3­(K131-L679)-Avi plasmid. For protein expression, 1.6 L of insect cells were cultured with a viral stock of the second passage for 48 h at 27 °C. Cells were collected, lysed in lysis buffer (50 mM HEPES pH 7.5, 500 mM NaCl, 10% glycerol, 1 mM MgCl_2_, 1 mM TCEP, 100 μM ATP) and centrifuged at 16,000 rpm for 60 min. The supernatant was loaded onto a Ni Bestarose FF column and eluted with lysis buffer containing 300 mM imidazole. The sample was biotinylated in a tube containing BirA enzyme (1:100 (w/w) to protein) with biotin buffer and TEV protease (1:10 (w/w) to protein), and the reaction was incubated at 4 °C overnight. The sample was collected and eluted with a lysis buffer containing 75 mM biotin from Strep-Tactin XT columns. The biotinylated NACHT protein was further purified with HiLoad 16/600 Superdex 200 pg column with an elution buffer (50 mM HEPES pH 7.5, 500 mM NaCl, 10% glycerol, 1 mM MgCl_2_, 1 mM TCEP).

### Immunoblotting

Protein samples were prepared with 4x Laemmli sample buffer (Bio-Rad) supplemented with β-mercaptoethanol, followed by boiling for 5–10 min. Samples were then resolved by SDS-PAGE and dry-transferred to poly­(vinyl difluoride) (PVDF) membranes using a Trans-Blot Turbo transfer system (Bio-Rad). Membranes were blocked in 5% (w/v) dried milk or BSA in TBS (Bio-Rad) supplemented with 0.1% (v/v) Tween-20 (TBS-T) for 1 h at room temperature or overnight at 4 °C. Membranes were then incubated with primary antibody, followed by a horseradish peroxidase (HRP)-conjugated secondary antibody, each diluted in 5% (w/v) dried milk or BSA in TBS-T for 1–2 h at room temperature or overnight at 4 °C. Membranes were developed with Clarity Western ECL substrate (Bio-Rad) and visualized for chemiluminescence using an Amersham Imager 680. Membranes can be stripped with stripping buffer (glycine (200 mM), SDS (3 mM), Tween-20 (1%), pH 2.2) before reprobing.

### Immunofluorescence Microscopy

J774A.1 cells were seeded onto poly-d-lysine-coated glass coverslips in a 12-well plate (1 × 10^6^ cells/well) and cultured in their respective growth medium, allowing them to attach overnight at 37 °C. For ASC speck formation, cells were treated with 1 μg/mL LPS for 4.5 h with different doses of compound treatments during the last hour, followed by treatment with 10 μM nigericin for 1.5 h. Cells were fixed with 4% paraformaldehyde, permeabilized with 0.1% Triton X-100 in PBS, and then blocked with 1% BSA and 0.1% Tween-20 in PBS. Subsequently, cells were treated with anti-ASC antibody (AL177, 1:200) overnight at 4 °C, followed by staining with Alexa Fluor 568-conjugated anti-rabbit secondary antibody (Thermo Fisher, 4 μg/mL). Nuclei were stained with DAPI (2 μg/mL), and coverslips were mounted onto slides using Epredia Immuno-Mount (Fisher Scientific). Images were acquired using a Zeiss LSM700 confocal microscope (Zeiss).

### Photoaffinity Labeling

30 μL aliquots of recombinant NLRP3 or a mixture of J774A.1 cell lysate and NLRP3 were incubated with or without YM-I-27 at the indicated concentrations at 37 °C for 30 min. The samples were then placed on ice and irradiated at 365 nm for 15 min. Subsequently, 1 μL of NBD-Azide (5 mM) or Biotin-PEG3-Azide (5 mM) was added to the samples, followed by the addition of 1 μL of CuSO_4_ (50 mM), tris­(benzyltriazolylmethyl)­amine (TBTA) (30 mM), and tris­(2-carboxyethyl)­phosphine (TCEP) (100 mM). The CuAAC reactions were agitated at room temperature for 1 h, and proteins were precipitated with prechilled methanol and left to dry. The protein pellets were then suspended in 30 μL of 2x SDS loading buffer and heated for 10 min at 95 °C. The SDS-PAGE gels of NBD-Azide-labeled proteins were then washed with washing buffer (10% acetic acid, 40% methanol in water) for 2 h and imaged using ChemiDoc MP Imaging System (Bio-Rad) with Alexa Fluor 488 (λ_ex_ = 490 nm, λ_em_ = 525 nm). The Biotin-PEG3-Azide-labeled proteins were then transferred to membranes and analyzed by Western blotting using streptavidin-HRP and NLRP3 antibodies, respectively.

### Fluorescence Resonance Energy Transfer (FRET) Assay

J774A.1 cells were treated with 1 μg/mL LPS for 4.5 h, and the photoactive probe (YM-I-27) was added and incubated for 30 min. Cells were irradiated at 365 nm for 30 min on ice and were fixed and permeabilized following the Immunofluorescence Microscopy procedures. The click reaction was performed in PBS containing TAMRA-N_3_ (30 μM), CuSO_4_ (800 μM), TBTA (500 μM), and TCEP (500 μM) at room temperature in the dark for 2.5 h. Cells were thoroughly washed with PBS and blocked by following the above procedures. Cells were then treated with anti-NLRP3 (cryo-2, 1:200) for 1.5 h, followed by Alexa Fluor 488-conjugated antimouse secondary antibody (Thermo Fisher, 4 μg/mL). Coverslips were mounted to slides, and data were acquired using a Zeiss LSM710 confocal microscope (Zeiss) and a 440 nm pulsed laser. The difference in the fluorescence lifetime was calculated by subtracting the median lifetime of Alexa Fluor 488 (donor) in the absence and presence of the YM-I-27 probe clicked with TAMRA (acceptor).

### Pull-Down Assay

LPS-primed J774A.1 cells were lysed with NP-40 lysis buffer (50 mM Tris-HCl at pH 8, 150 mM NaCl, 1.0% NP-40, supplemented with cOmplete mini protease inhibitor cocktail) and centrifuged at 20,000*g* for 20 min at 4 °C. The supernatant was diluted to a concentration of 1.2 mg/mL and incubated with the competing compound for 1 h on ice, followed by incubation with YM-I-85 for 1 h on ice. Samples were irradiated at 365 nm for 15 min and then incubated with Streptavidin Agarose Resin (Thermo Fisher) overnight at 4 °C. The resins were washed once with 0.1% SDS in PBS and twice with 1% NP-40 in PBS before eluting with SDS-PAGE loading buffer and boiling. Beads were spun down, and the supernatants were analyzed by immunoblotting.

### Cellular Thermal Shift Assay (CETSA)

Expi293F cells were seeded into T75 culture flasks and transfected with pcDNA3-FLAG-mNLRP3 (Addgene #75127) to express FLAG-NLRP3. Then, the cells were treated with YQ128 for 1 h, washed, and resuspended in PBS supplemented with a complete mini protease inhibitor cocktail, and aliquoted into PCR strips at 3 million cells per tube. PCR strips were heated to the respective temperatures for 3 min using a thermocycler and then cooled at room temperature for 3 min. Cells were lysed by freeze–thaw, and debris was spun down at 20,000*g* for 20 min at 4 °C. Supernatants were analyzed by immunoblotting.

### Microscale Thermophoresis (MST)

The binding affinity was measured with a Monolith NT.Automated instrument as described previously.^61^ Recombinant human NLRP3 protein (500 nM) was labeled with 50 nM RED-tris-NTA dye (NanoTemper Technologies, MO-L018). After centrifugation, the supernatant was aliquotted and incubated with a range of concentrations of YQ128 for 40 min in PBS-T assay buffer (1x PBS with 0.05% Tween 20). The samples were loaded into NanoTemper glass capillaries, and thermophoresis curves were analyzed using the instrument. *K*_D_ values are representative of independent experiments (*n* = 3).

### Surface Plasmon Resonance (SPR) Experiments

SPR experiments were performed on a Biacore 1K system with streptavidin sensor chip (Series S Sensor Chip SA, Cytiva) according to the reported procedure.^26^ The system was flushed with running buffer (10 mM HEPES, pH 7.4, 150 mM NaCl, 0.5 mM ADP, 0.5 mM TCEP, 2 mM MgCl_2_, 0.05% Tween 20, 2% DMSO). The SA chips were conditioned with 1 M NaCl in 50 mM NaOH (1 min, 10 μL/min) three times. Biotinylated NACHT NLRP3 protein (50 μg/mL) was loaded onto the sensor surfaces at a rate of 10 μL/min for 600 s, followed by blockage for residual streptavidin binding sites with Biotin-PEG (40 μM) for 2 min at 10 μL/min. After equilibration for 2 h, various concentrations of compounds in running buffer were tested in a single-cycle kinetics mode (30 μL/min). Data were collected at a rate of 10 Hz and fitted to a 1:1 interaction model using the Biacore Insight Evaluation Software.

### Drug Affinity Responsive Target Stability (DARTS)

DARTS assay was performed with full-length human NLRP3, human PYD domain (1–100aa) (Abnova, H00114548-Q01), and human PYD-NACHT domain (1–536aa) (CUSABIO, CSB-EP822275HU1). A 2 μg amount of protein was incubated with or without YQ128 (50 μM) on ice for 2–3 h. Then, 0.1 mg/mL Pronase (Sigma-Aldrich) was added at the protease to protein ratio, between 50 and 200 ng Pronase per μg of protein for 7.5–30 min at room temperature. The reaction was stopped by the addition of a 20x protease inhibitor cocktail and incubated on ice for 10 min. Protein samples were analyzed by immunoblotting.

### Dot Blot Assay

The assay was performed with 1–2 μg of FL-NLRP3 in 20 μL of assay buffer (20 mM Tris-HCl, 150 mM NaCl, 10% glycerol, pH 7.5). Then, 0.2 μL of YQ128 and MCC950 were sequentially added at 100x final concentration in DMSO (final concentrations: 0.3, 3, 30 μM) and incubated at room temperature for 1 h. 2 μL of the sample was spotted onto the nitrocellulose membrane (vertical) using a narrow-mouth pipette tip. The membrane was dried, and the samples were processed using the immunoblotting procedure.

### Fluorescence Polarization (FP) Assay

The assays were conducted following previously described procedures^58^ using the NLRP3 NanoBRET tracer from the NLRP3 NanoBRET Target Engagement Assay kit^57^ in an assay buffer (50 mM HEPES (pH 7.4), 150 mM NaCl, 2.5 mM MgCl_2_, 0.005% Tween 20, 1 mM TCEP, 100 μM ATP) and measured accordingly. Briefly, a 100 nM tracer was first used to titrate the recombinant NACHT protein (*K*_d_ = 700 nM). Then, 700 nM NACHT was used to titrate the tracer (*K*_d_ = 5 nM). For the competition, NACHT protein was diluted to 700 nM in the assay buffer and incubated at room temperature for 1 h with varying concentrations of MCC950 or YQ128. Then, the NLRP3 NanoBRET tracer (5 nM) was added to each well and incubated for another hour, covered, prior to measuring the fluorescence polarization (λ_ex_ = 550 nm, λ_em_ = 600 nm). Data represent the average from three independent experiments.

### Molecular Docking and Molecular Modeling

Glide model from Schrödinger software was used for the molecular docking studies. The structures of the compounds were sketched using ChemDraw and optimized to lower-energy conformers using Ligprep (Schrödinger LLC). The structure of human NLRP3 (PBD ID: 7PZC) was downloaded from the Protein Data Bank, and a Protein Preparation Wizard was used to prepare the complex for docking. OPLS3 force field was used to optimize the hydrogen bond network in the protein structure after a series of preprocessing, such as mutation, adding hydrogens, deleting water, etc. A maximum cutoff of 0.30 Å was applied for energy convergence or the root mean square deviation (RMSD). The receptor grid was generated using the Receptor Grid Generation panel. Ultimately, the generated grid of the protein structure was used to perform docking with the compounds using Ligand Docking under the XP (extra precision) mode. The Glide docking score was used as the evaluation standard for ligand-protein binding.

### Statistical Analysis

The values are expressed as mean ± SD. The unpaired *t* test (GraphPad Software) was used for statistical analysis. The data points were not excluded. *p < 0.05, **p < 0.01, ***p < 0.001. *P*-values <0.05 were considered significant. *ns* means not significant.

## Supplementary Material





## Notes

Synthesis, HPLC and NMR spectra for compounds YM-I-27 and YM-I-85; additional biological data of YQ128 (PDF)
Molecular formula strings (CSV)

## Abbreviations

ASC: apoptosis-associated speck-like protein containing a caspase-1 recruitment domain
ATP: adenosine triphosphate
CETSA: cellular thermal shift assay
DAMPs: damage-associated molecular patterns
DARTS: drug affinity responsive target stability
DTT: dithiothreitol
FP: fluorescence polarization
FRET: fluorescence resonance energy transfer
GSDMD: gasdermin D
HRP: horseradish peroxidase
IL-1β and IL-18: interleukin-1β and interleukin-18
LPS: lipopolysaccharide
MOAs: mechanisms of action
MST: microscale thermophoresis
NLRP3: NOD-like receptor protein 3
NEK7: NIMA-related kinase 7
NBD: nitrobenzoxadiazole
PAMPs: pathogen-associated molecular patterns
PAL: photoaffinity labeling
PYD: pyrin domain
PVDF: polyvinyl difluoride
SAR: structure–activity relationship
SPR: surface plasmon resonance
TNF-α: tumor necrosis factor
TBTA: tris­(benzyltriazolylmethyl)­amine
TCEP: tris­(2-carboxyethyl)­phosphine
