# Supplementary material for: Mechanistic Characterization of a Small Molecule as a Direct NLRP3 Inhibitor via Binding to the NACHT Domain
Source: J Med Chem. 2025 Nov 12;68(22):24260–71. doi: 10.1021/acs.jmedchem.5c02163 (PMC12670425; doi:10.1021/acs.jmedchem.5c02163)
Supplement: Supplementary file 1 [file jm5c02163_si_001.pdf]

Mechanistic characterization of a small molecule as direct NLRP3 inhibitor via binding to the NACHT domain

Yiming Xu[a], Hallie Blevins[a], Savannah Biby[a], Jannatun N. Namme[a], Kun Zhang[b], Renfeng Li[c], Shijun Zhang[a*]

[a]*Department of Medicinal Chemistry, School of Pharmacy,* [b]*Philips Institute for Oral Health Research, School of Dentistry, Virginia Commonwealth University, Richmond, Virginia 23298, United States*

[c]*Department of Microbiology and Molecular Genetics, University of Pittsburgh, Pittsburgh, Pennsylvania 15232, United States*

*Corresponding Author:

Shijun Zhang, Ph.D., Tel: 804-6288266, Fax: 804-8287625, E-mail: szhang2@vcu.edu

ORCID: 0000-0001-9732-5925

Content of SI

SI1: Chemical Synthesis, Page S2-S9

SI2: Biological data, Page S10-S12

SI3: References, Page S13

SI1: Chemical Synthesis

Scheme 1. Synthesis of diazirine-alkyne (**1-7**), the detailed methods were reported previously.[1]

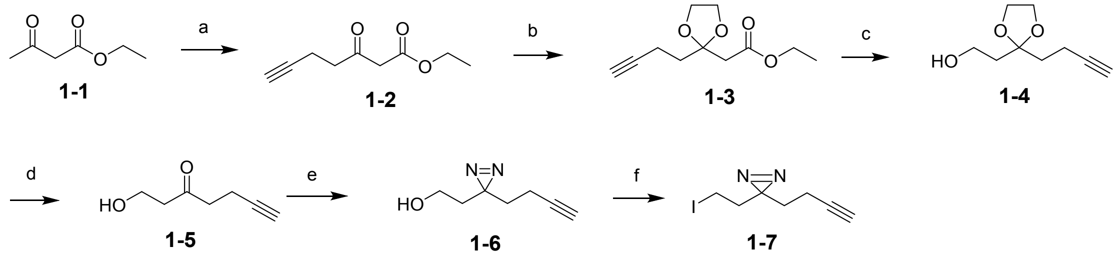

Reagents and conditions: (a) propargyl bromide, LDA, -78 °C to rt; (b) ethylene glycol, benzene, reflux; (c) $LiAlH_4$, THF; (d) TsOH, acetone; (e) NH_3, NH_2SO_3H; I_2, TEA; (f) PPh_3, I_2, imidazole, THF.

Scheme 2. Synthesis of Biotin-PEG2-N_3 (**2-7**), the detailed methods were reported previously.[2, 3]

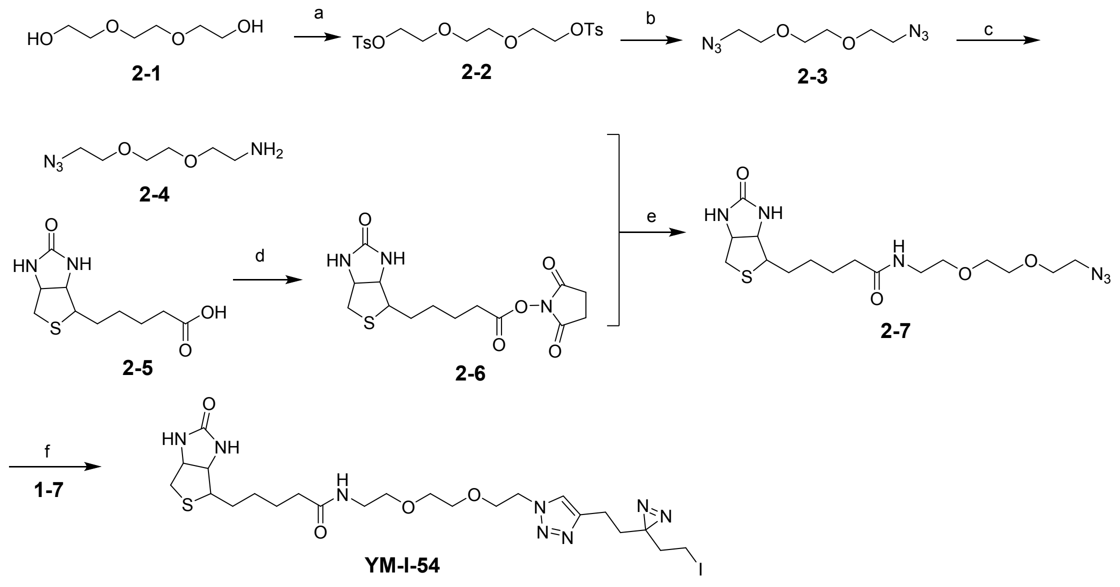

Reagents and conditions: (a) NaOH, TsCl, DCM; (b) NaN_3, TBAI, DMF, 80 °C; (c) PPh_3, HCl, rt; (d) NHS, DCC, DMF, rt; (e) DIPEA, DMF, rt; (f) $CuSO_4$, sodium ascorbate, H_2O/THF, rt.

Compounds **2-7** (344 mg, 0.86 mmol), **1-7** (213 mg, 0.86 mmol), $CuSO_4$•$5H_2O$ (11 mg, 0.043 mmol) and sodium ascorbate (17 mg, 0.086 mmol) were dissolved in a mixture of H_2O/THF (6 mL, 1:2, v/v). The mixture was stirred at r.t. for 24 h. H_2O (10 mL) was added to the reactants followed by extraction with dichloromethane (3 × 5 mL). The combined organic layers were washed with brine (2 × 10 mL), dried over anhydrous Na_2SO_4 and concentrated under vacuum. The crude product was purified by flash chromatography (Chloroform/MeOH=6:1) to afford **YM-I-54** (435 mg, yield: 78%) as

yellow oil.

YM-I-54. ^{1}H NMR (400 MHz, Chloroform-d) δ 7.52 (d, J = 4.2 Hz, 1H), 6.45 (s, 1H), 4.56 – 4.39 (m, 3H), 4.28 (m, 1H), 3.84 (t, J = 5.2 Hz, 2H), 3.70 – 3.22 (m, 9H), 3.09 (m, 1H), 2.83 (m, 3H), 2.69 (m, 1H), 2.60 – 2.33 (m, 2H), 2.18 (m, 2H), 2.01 (t, J = 7.3 Hz, 2H), 1.94 – 1.80 (m, 2H), 1.80 – 1.53 (m, 4H), 1.40 (m, 2H). ^{13}C NMR (101 MHz, $CDCl_3$) δ 173.36, 163.53, 145.50, 122.85, 77.22, 70.47, 70.19, 69.87, 69.34, 61.95, 60.32, 55.42, 50.61, 40.49, 39.20, 37.48, 35.74, 31.85, 28.80, 28.06, 28.02, 25.56, 19.67. HRMS (APESI) m/z: calcd for $C_{23}H_{37}IN_8O_4S$ $[M + Na]^+$, 671.1595; found, 671.1559.

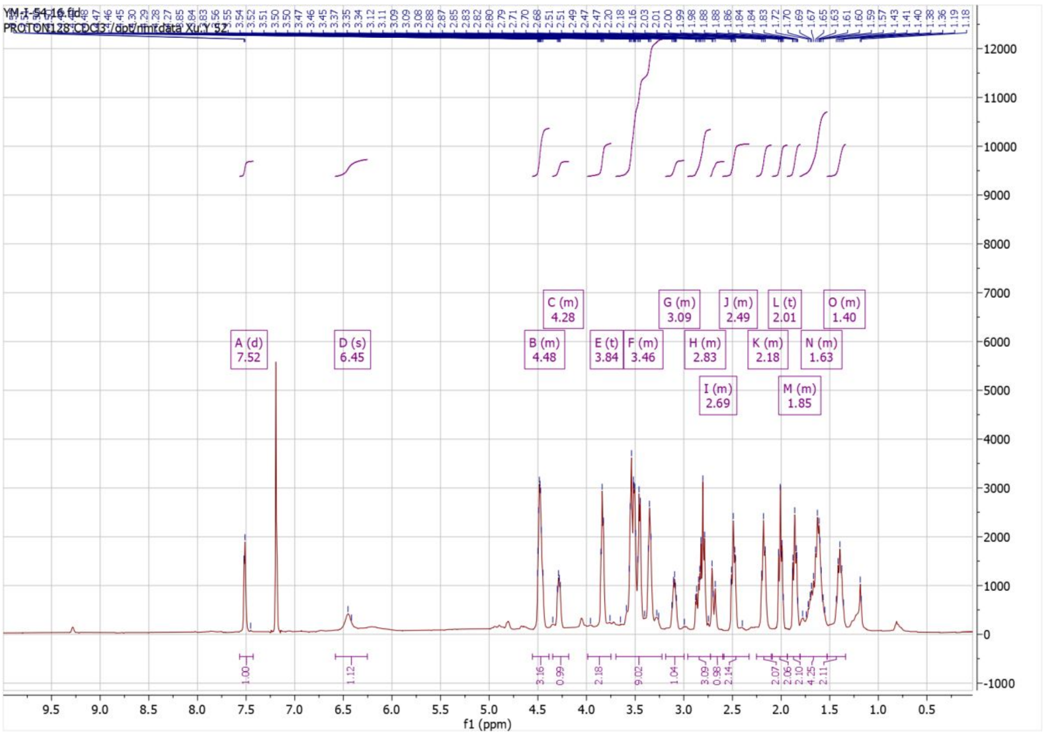

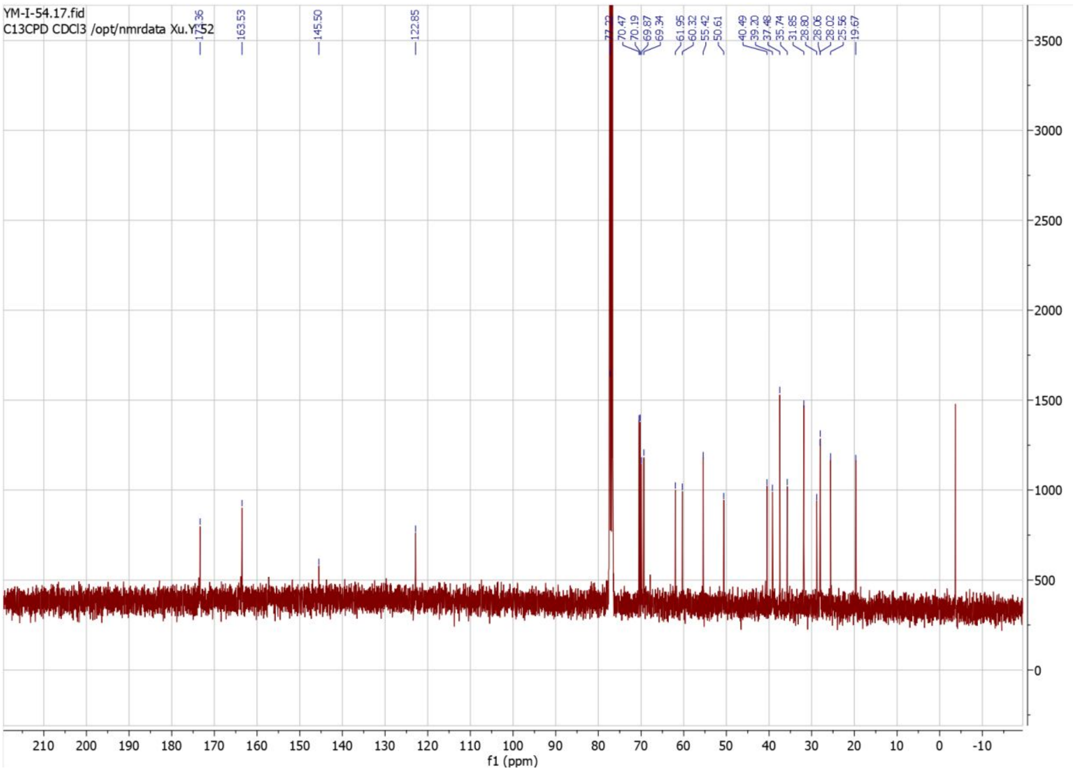

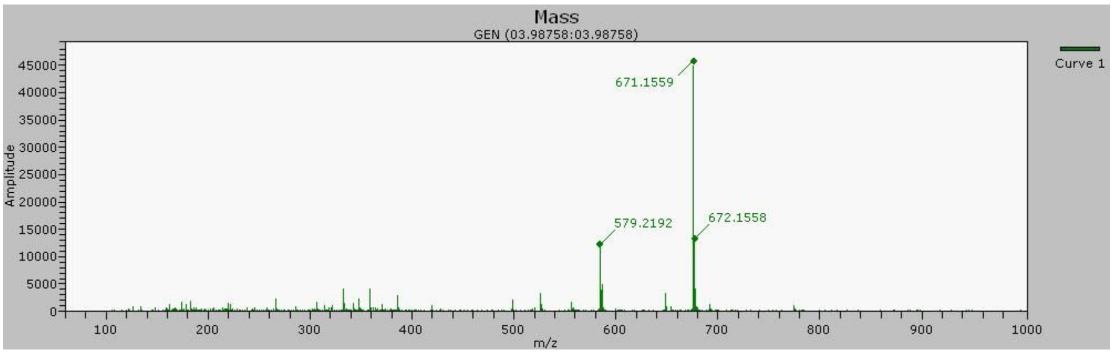

Scheme 3. Synthesis of **YM-I-12**, the detailed methods were reported previously.[4]

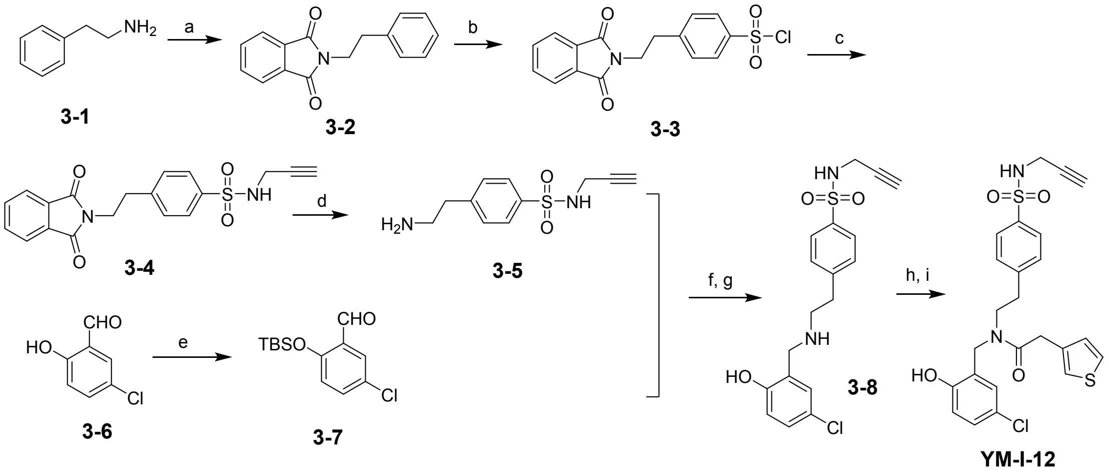

Reagents and conditions: (a) phthalic anhydride, AcOH, reflux; (b) $ClSO_3H$, 70 °C; (c) propargylamine, TEA, DCM; (d) NH_2NH_2, EtOH, 60 °C; (e) TBSCl, imidazole,

DCM; (f) Acetic acid, $NaCNBH_3$; (g) 1M TBAF, THF (h) 3-Thiopheneacetic acid, EDCI, HOBt, TEA, DCM; (i) K_2CO_3, MeOH.

Scheme 4. Synthesis of **YM-I-85** and **YM-I-27** probes.

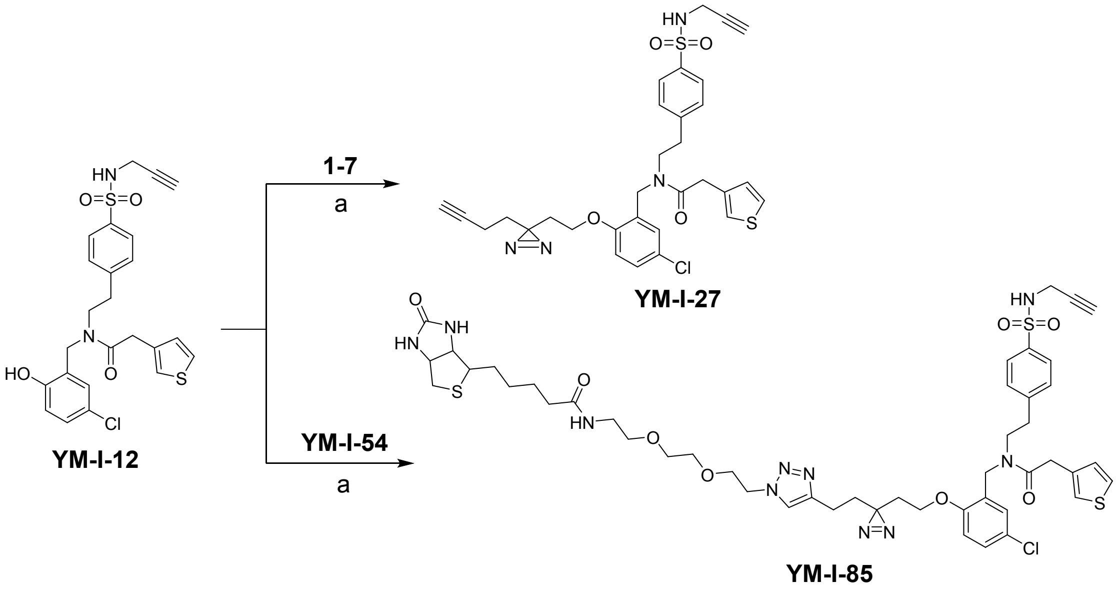

Reagents and conditions: (a) Cs_2CO_3, acetone, reflux, overnight.

Compounds **YM-I-12** (150 mg, 0.30 mmol), **1** (50 mg, 0.20 mmol) or **YM-I-54** (130 mg, 0.20 mmol), and cesium carbonate (130 mg, 0.4 mmol) were mixed with acetone (5 mL). The mixture was stirred and refluxed overnight. After completion of the reaction, acetone was removed and residue was partitioned between dichloromethane and water. The dichloromethane layer was washed with brine, dried over anhydrous Na_2SO_4 and concentrated under vacuum. The crude product was purified by flash chromatography with hexane/acetate (2:1) to give **YM-I-27** (72 mg, yield: 58%) or Chloroform/MeOH (6:1) to give **YM-I-85** (98 mg, yield: 48%) as colorless oil.

YM-I-27. ^{1}H NMR (400 MHz, DMSO-d_6) δ 8.00 (m, 1H), 7.65 (m, 2H), 7.51 – 7.06 (m, 5H), 7.00 – 6.74 (m, 3H), 4.49 (d, J = 7.5 Hz, 2H), 3.79 (m, 2H), 3.70 – 3.35 (m, 6H), 3.09 – 2.55 (m, 4H), 1.95 (m, 2H), 1.87 – 1.72 (m, 2H), 1.58 (m, 2H). ^{13}C NMR (101 MHz, DMSO-d_6) δ 170.86, 155.09, 143.94, 136.09, 130.03, 129.72, 129.15, 128.94, 127.35, 127.28, 126.43, 124.88, 122.90, 122.76, 113.69, 83.65, 79.82, 75.05, 72.25, 63.46, 49.23, 43.13, 34.79, 32.37, 32.21, 32.06, 32.02, 27.50, 13.12. HRMS (APESI) m/z: calcd for $C_{31}H_{31}ClN_4O_4S_2$ $[M + Na]^+$, 645.1367; found, 645.1337.

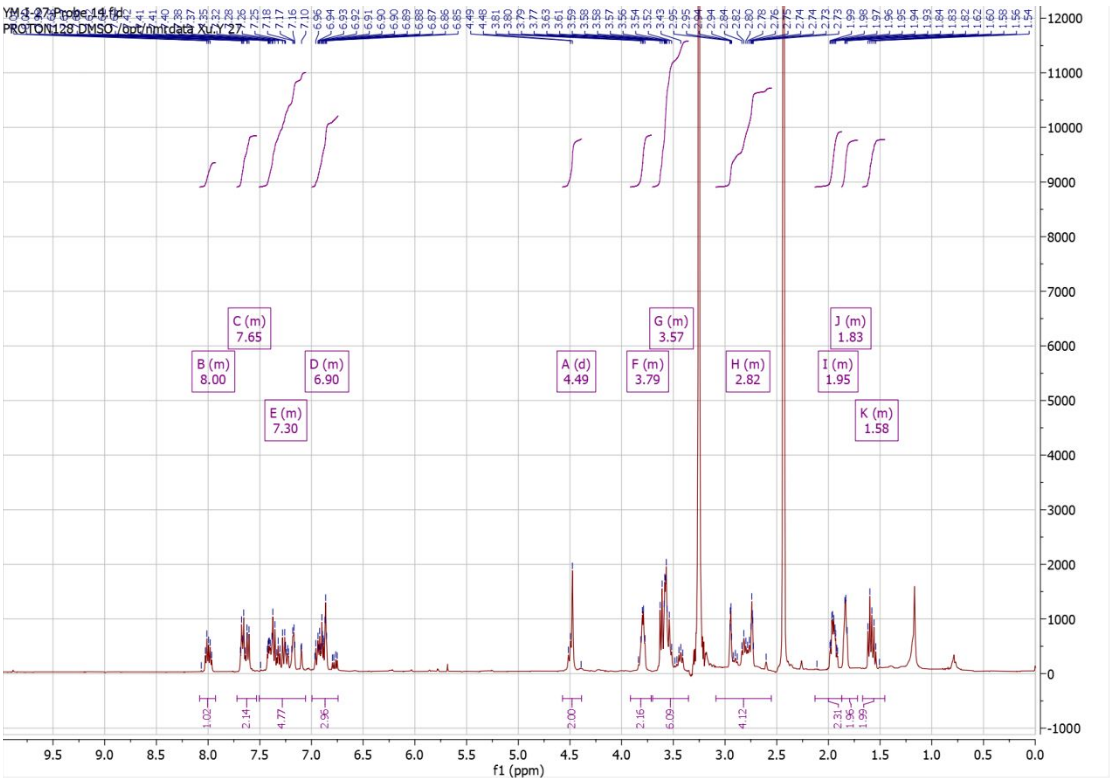
YM-I-27-Probe.14.fid
PROTON128 DMSO /opt/nmrdata Xu.Y 27
A (d) 4.49
B (m) 8.00
C (m) 7.65
D (m) 6.90
E (m) 7.30
F (m) 3.79
G (m) 3.57
H (m) 2.82
I (m) 1.95
J (m) 1.83
K (m) 1.58
f1 (ppm)

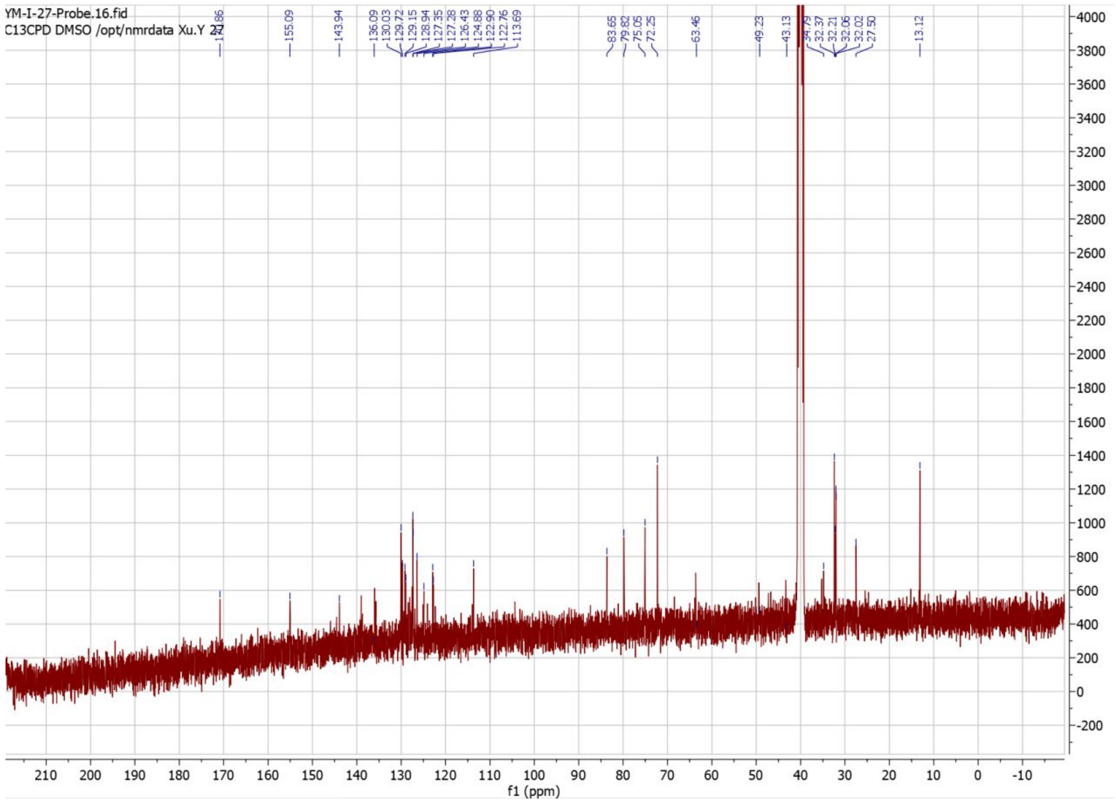
YM-I-27-Probe.16.fid
C13CPD DMSO /opt/nmrdata Xu.Y 27
155.09
143.94
136.09
130.03
129.72
129.15
128.94
127.35
127.28
126.43
124.88
122.90
122.76
113.69
83.65
79.82
75.05
72.25
63.46
49.23
43.13
32.37
32.21
32.06
32.02
27.50
13.12
f1 (ppm)

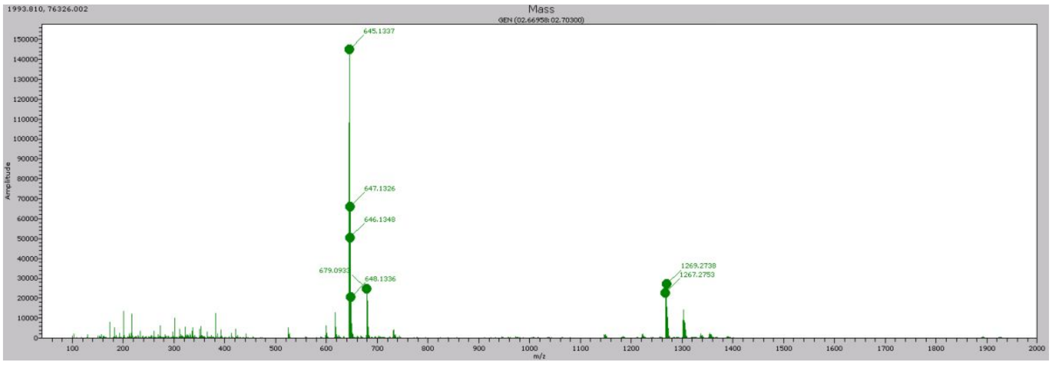

HPLC trace of YM-I-27

HPLC conditions
Column: SunFire C18 250 × 4.6 mm column
UV detection (254 nm)
Solvent: 70% acetonitrile/ H_2O with 0.1% trifluoroacetic acid (TFA)
Flow rate: 1 mL/min

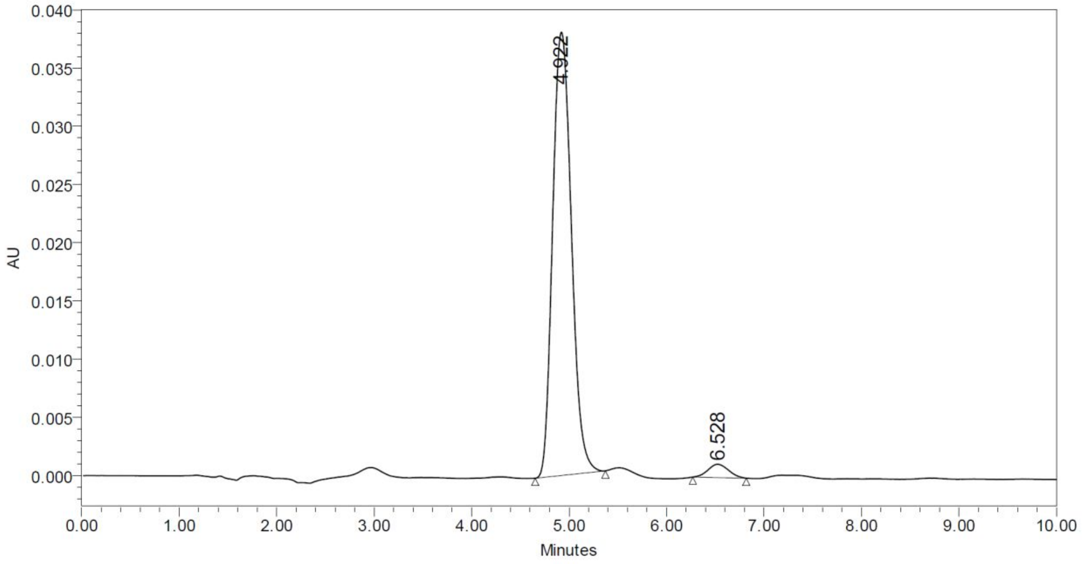

	RT	Area	% Area	Height
1	4.922	507936	96.81	38073
2	6.528	16724	3.19	1139

YM-I-85.
^{1}H NMR (400 MHz, DMSO-*d6*) δ 8.03 (m, 1H), 7.88 – 7.62 (m, 4H), 7.62 – 7.39 (m, 2H), 7.39 – 7.05 (m, 3H), 7.05 – 6.87 (m, 3H), 6.37 (m), 5.76 (s, 1H), 4.63 – 4.36 (m, 4H), 4.30 (dd, J = 7.7, 5.1 Hz, 1H), 4.19 – 4.04 (m, 1H), 3.91 – 3.55 (m, 9H), 3.55 – 3.42 (m, 6H), 3.24 – 2.99 (m, 5H), 2.98 – 2.73 (m, 3H), 2.58 (m, 1H), 2.49 – 2.35 (m, 2H), 2.06 (t, J = 7.2 Hz, 2H), 1.96 – 1.72 (m, 3H), 1.72 – 1.55 (m, 1H), 1.56 – 1.40 (m, 3H), 1.29 (m, 3H). ^{13}C NMR (101 MHz, DMSO-d_6) δ 172.58, 170.86, 163.16, 155.09, 145.66, 143.94, 139.00, 135.98, 130.03, 129.71, 129.16, 128.41, 128.03, 127.34,

127.27, 126.43, 124.87, 122.87, 113.65, 79.82, 75.07, 75.05, 69.98, 69.85, 69.60, 69.25, 69.23, 63.67, 61.50, 59.65, 55.88, 55.37, 49.71, 43.36, 38.87, 35.56, 34.79, 33.35, 32.50, 32.37, 28.65, 28.50, 27.72, 25.71, 20.03. HRMS (APESI) m/z: calcd for $C_{47}H_{59}ClN_{10}O_8S_3$ $[M + Na]^+$, 1045.3260; found, 1045.3231.

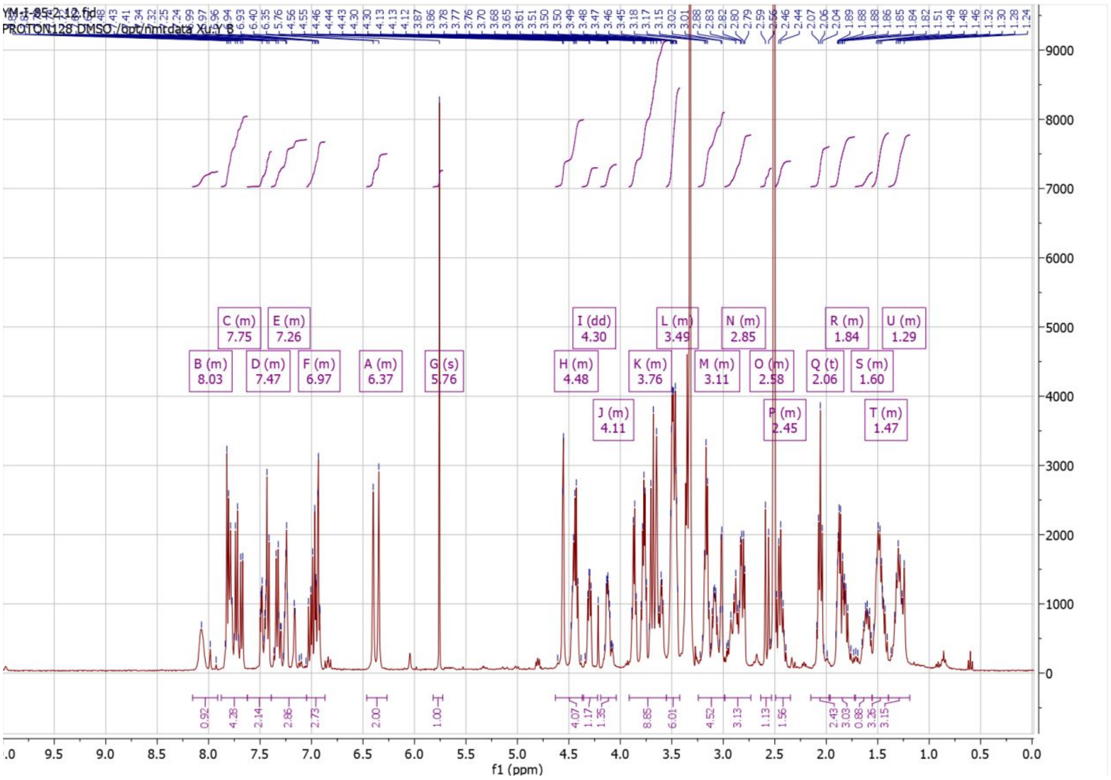

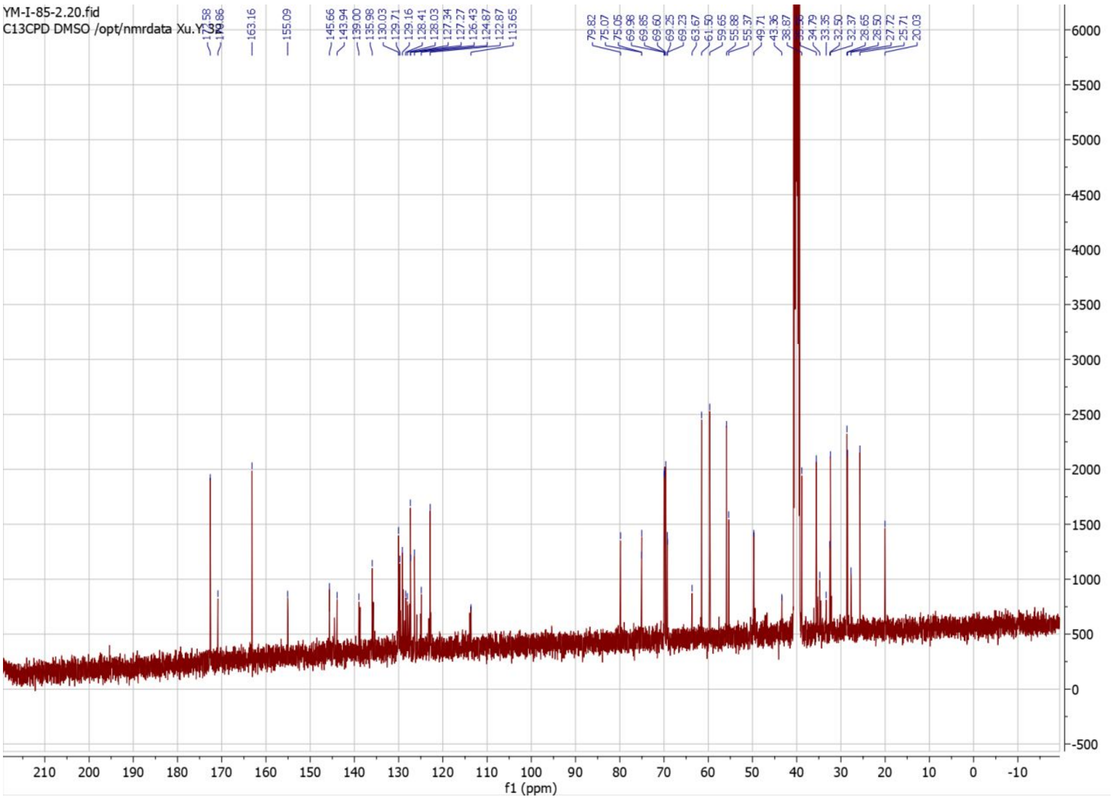

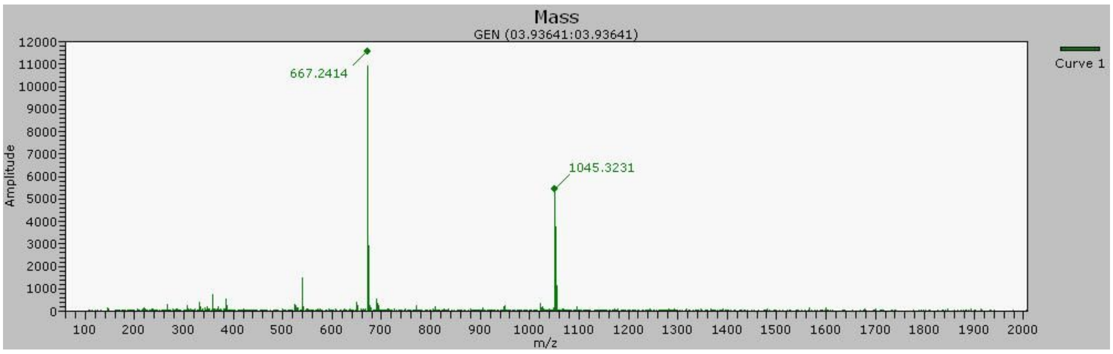

HPLC trace of YM-I-85
HPLC conditions
Column: SunFire C18 250 × 4.6 mm column
UV detection (254 nm)
Solvent: 50% MeOH/ H_2O with 0.1% trifluoroacetic acid (TFA)
Flow rate: 1 mL/min

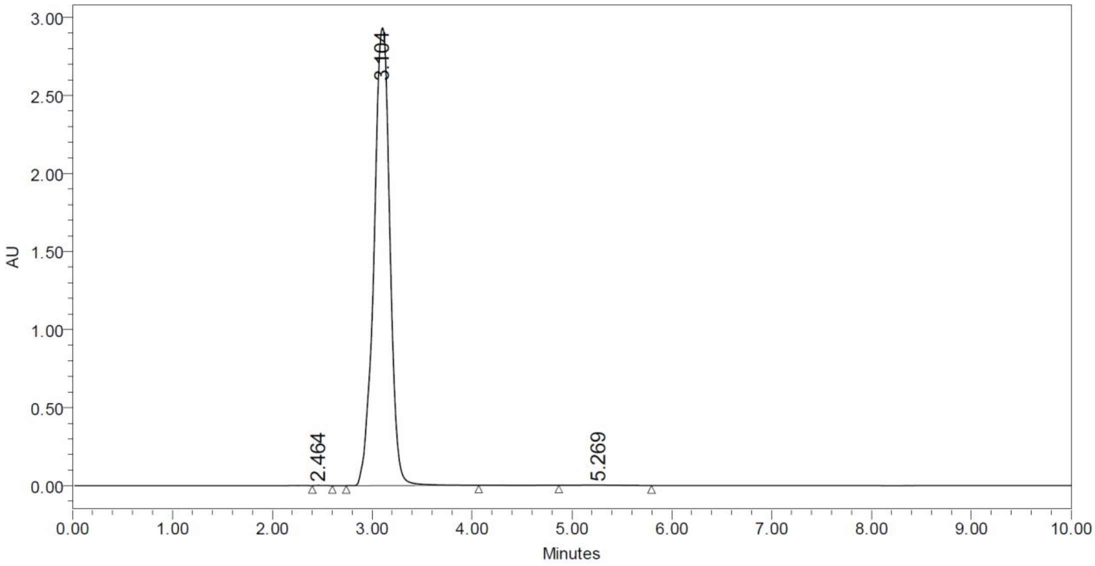

	RT	Area	% Area	Height
1	2.464	5550	0.02	1072
2	3.104	32580475	99.84	2931529
3	5.269	45829	0.14	1629

SI2: Biological Data

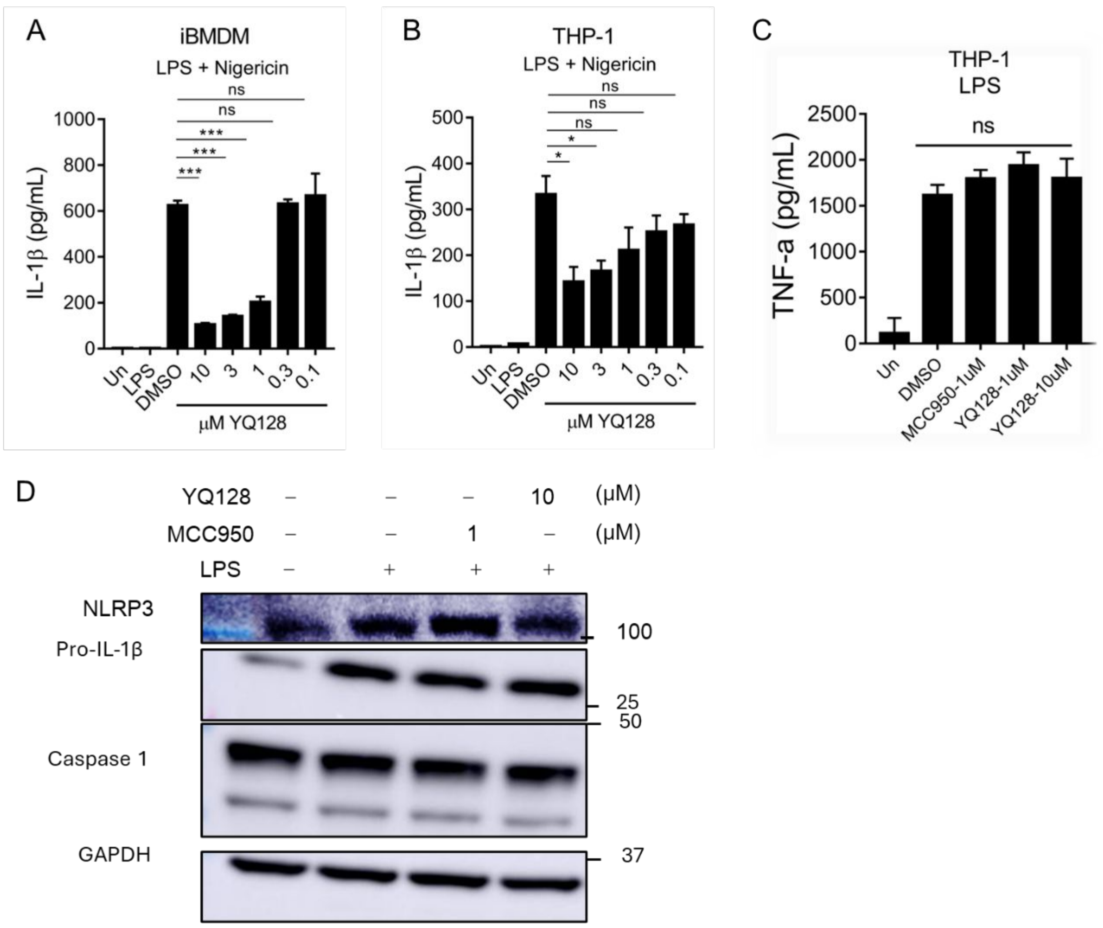

Figure S1. (A) iBMDMs were primed with LPS (1 µg/mL) for 3 h and treated with or without YQ128 for 30 min, followed by addition of nigericin (10 µM) for 2 h. (B) THP-1 cells were differentiated into adherent macrophages by culturing for 24 h in complete RPMI 1640 medium containing 50 nM PMA followed by 24 h resting period. The differentiated THP-1 cells were primed with LPS (1 µg/mL) for 2.5 h and treated with or without YQ128 for 30 min, followed by addition of nigericin (10 µM) for 1 h. The levels of IL-1β and TNF-α in supernatant were measured by ELISA. (C) Western blotting analysis of NLRP3, pro-IL-1β and caspase 1 in cell lysates from differentiated THP-1 cells treated with YQ128 (10 µM) or MCC950 (1 µM) and stimulated with LPS (1 µg/mL). Statistical analysis by unpaired Student's t-test: *P < 0.05, **P < 0.01, ***P < 0.001, ns means not significant.

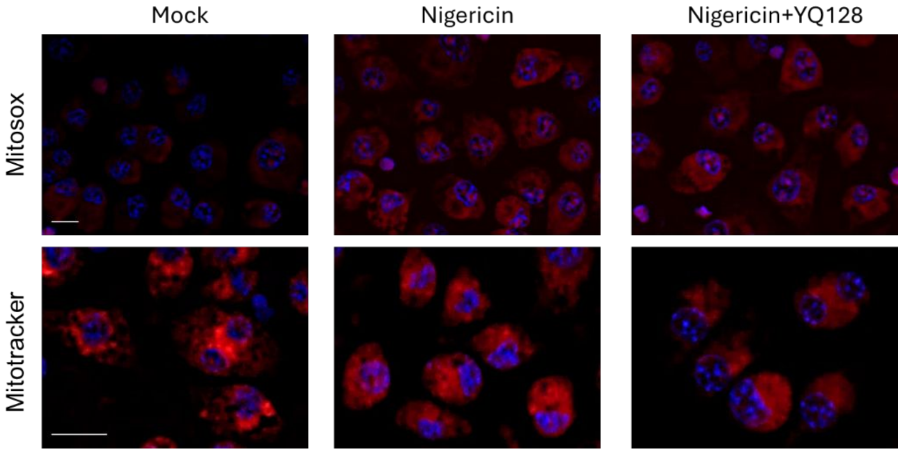

Figure S2. J774A.1 were primed with LPS (1 μg/mL) for 4.5 h and treated with or without YQ128 (10 μM) for 30 min, followed by addition of nigericin (3 μM) for 1.5 h. Cells were stained with MitoSOX and MitoTracker red. Nuclei were stained with DAPI. Bars, 10 μm.

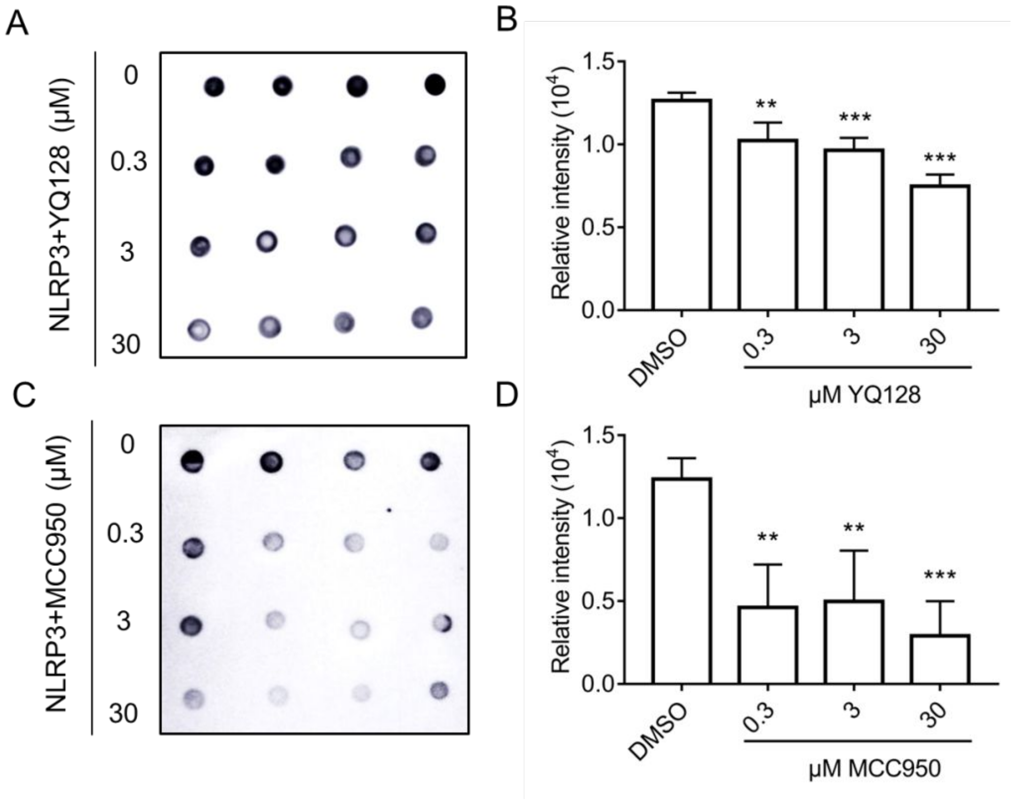

Figure S3. YQ128 (A&B) and MCC950 (C&D) dose-dependently block the recognition of NLRP3 by D4D8T antibody.

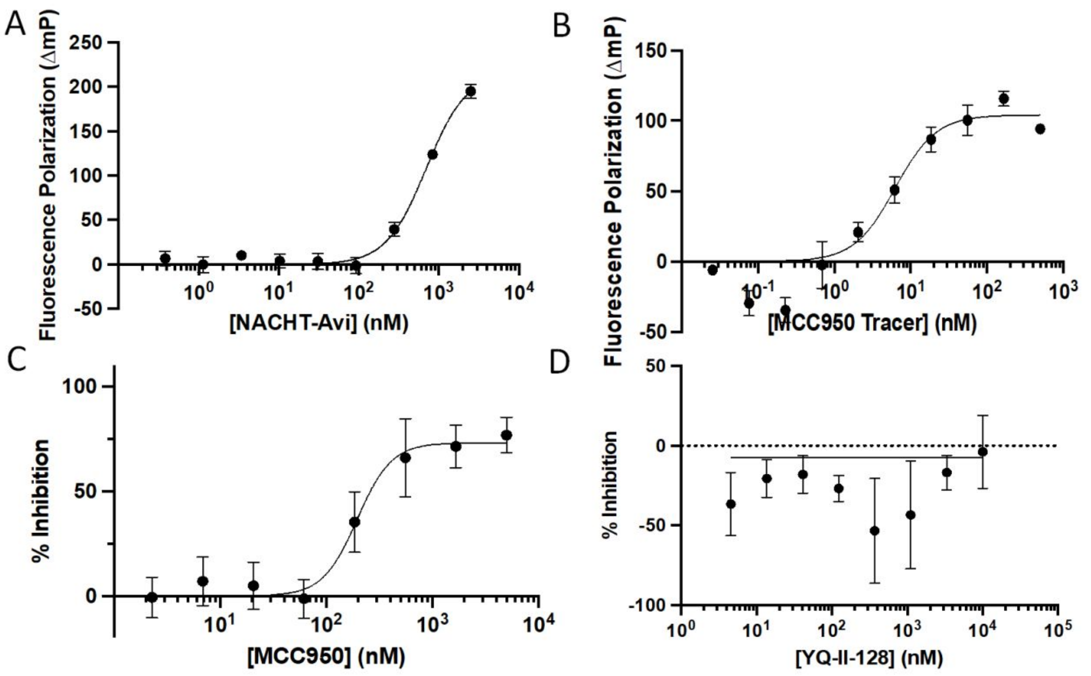

Figure S4. (A) Titration of the NACHT protein in the presence of NLRP3 NanoBRET® tracer (100 nM). (B) Titration of the tracer in the presence of NACHT protein (700 nM). MCC950 (C) or YQ128 (D) was added with the NanoBRET® tracer (5 nM) to NACHT protein (700 nM) and the change of fluorescence polarization was recorded and calculated. The data is an average of three independent experiments.

SI3: References

(1) Li, Z.; Hao, P.; Li, L.; Tan, C. Y.; Cheng, X.; Chen, G. Y.; Sze, S. K.; Shen, H. M.; Yao, S. Q. Design and synthesis of minimalist terminal alkyne-containing diazirine photo-crosslinkers and their incorporation into kinase inhibitors for cell- and tissue-based proteome profiling. *Angew. Chem. Int. Ed. Engl.* **2013**, *52* (33), 8551-8556.
(2) Klein, E.; Kerth, P.; Lebeau, L. Enhanced selective immobilization of biomolecules onto solid supports coated with semifluorinated self-assembled monolayers. *Biomater.* **2008**, *29* (2), 204-214.
(3) Bonger, K. M.; van den Berg, R. J.; Heitman, L. H.; AP, I. J.; Oosterom, J.; Timmers, C. M.; Overkleeft, H. S.; van der Marel, G. A. Synthesis and evaluation of homo-bivalent GnRHR ligands. *Bioorg. Med. Chem.* **2007**, *15* (14), 4841-4856.
(4) Xu, Y.; Blevins, H.; Lan, Y.; Liu, Y.; Yuan, G.; Striar, R.; Zagaroli, J. S.; Tocci, D. R.; Langan, A. G.; Zhang, C.; et al. Discovery of carbon-11 labeled sulfonamide derivative: A PET tracer for imaging brain NLRP3 inflammasome. *Bioorg. Med. Chem. Lett.* **2021**, *34*, 127777.
